# Robustness of radiomics features of virtual unenhanced and virtual monoenergetic images in dual-energy CT among different imaging platforms and potential role of CT number variability

**DOI:** 10.1186/s13244-023-01426-5

**Published:** 2023-05-11

**Authors:** Jingyu Zhong, Zilai Pan, Yong Chen, Lingyun Wang, Yihan Xia, Lan Wang, Jianying Li, Wei Lu, Xiaomeng Shi, Jianxing Feng, Fuhua Yan, Huan Zhang, Weiwu Yao

**Affiliations:** 1 Department of Imaging, Tongren Hospital, Shanghai Jiao Tong University School of Medicine, Shanghai, 200336 China; 2 Department of Radiology, Ruijin Hospital, Shanghai Jiao Tong University School of Medicine, Shanghai, 200025 China; 3 Computed Tomography Research Center, GE Healthcare, Beijing, 100176 China; 4 Computed Tomography Research Center, GE Healthcare, Shanghai, 201203 China; 5 Department of Materials, Imperial College London, London, SW7 2AZ UK; 6 Haohua Technology Co., Ltd., Shanghai, 201100 China

**Keywords:** Machine learning, Multidetector computed tomography, Reproducibility of results, Image enhancement, Image reconstruction

## Abstract

**Objectives:**

To evaluate robustness of dual-energy CT (DECT) radiomics features of virtual unenhanced (VUE) image and virtual monoenergetic image (VMI) among different imaging platforms.

**Methods:**

A phantom with sixteen clinical-relevant densities was scanned on ten DECT platforms with comparable scan parameters. Ninety-four radiomic features were extracted via Pyradiomics from VUE images and VMIs at energy level of 70 keV (VMI_70keV_). Test–retest repeatability was assessed by Bland–Altman analysis. Inter-platform reproducibility of VUE images and VMI_70keV_ was evaluated by coefficient of variation (CV) and quartile coefficient of dispersion (QCD) among platforms, and by intraclass correlation coefficient (ICC) and concordance correlation coefficient (CCC) between platform pairs. The correlation between variability of CT number radiomics reproducibility was estimated.

**Results:**

92.02% and 92.87% of features were repeatable between scan–rescans for VUE images and VMI_70keV_, respectively. Among platforms, 11.30% and 28.39% features of VUE images, and 15.16% and 28.99% features of VMI_70keV_ were with CV < 10% and QCD < 10%. The average percentages of radiomics features with ICC > 0.90 and CCC > 0.90 between platform pairs were 10.00% and 9.86% in VUE images and 11.23% and 11.23% in VMI_70keV_. The CT number inter-platform reproducibility using CV and QCD showed negative correlations with percentage of the first-order radiomics features with CV < 10% and QCD < 10%, in both VUE images and VMI_70keV_ (*r*^2^ 0.3870–0.6178, all *p* < 0.001).

**Conclusions:**

The majority of DECT radiomics features were non-reproducible. The differences in CT number were considered as an indicator of inter-platform DECT radiomics variation.

**Critical relevance statement:** The majority of radiomics features extracted from the VUE images and the VMI70keV were non-reproducible among platforms, while synchronizing energy levels of VMI to reduce the CT number value variability may be a potential way to mitigate radiomics instability.

**Graphical Abstract:**

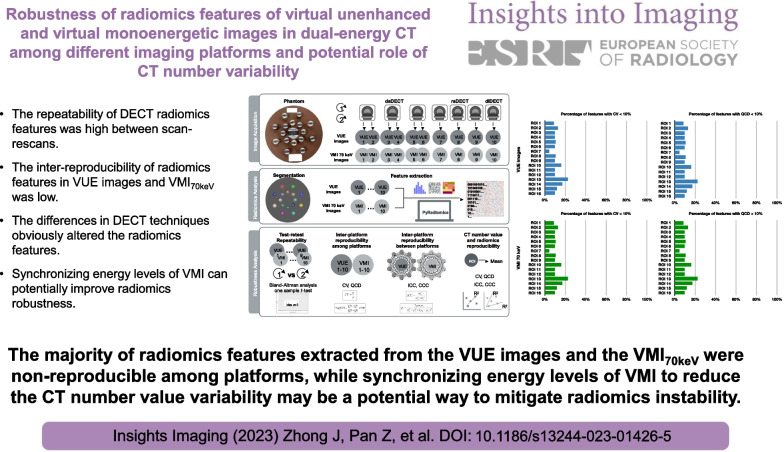

**Supplementary Information:**

The online version contains supplementary material available at 10.1186/s13244-023-01426-5.

## Key points

- The repeatability of DECT radiomics features was high between scan–rescans.
- The inter-reproducibility of radiomics features in VUE images and VMI_70keV_ was low.
- The differences in DECT techniques obviously altered the radiomics features.
- Synchronizing energy levels of VMI can potentially improve radiomics robustness.

**Supplementary Information:**

The online version contains supplementary material available at 10.1186/s13244-023-01426-5.

## Introduction

Radiomics extracts minable data from medical images to answer diagnostic, prognostic, and predictive questions, with the aim to deliver precision medicine [1–5]. Although numerous studies have shown its potential for clinical decision-making, gap between promising the academic results and the clinical utilization still exists due to instability of radiomics features [6–9]. The robustness of radiomics features has been demonstrated to be sensitive and fragile to variations of data acquisition, image reconstruction, segmentation, image processing, and radiomics feature computation. The standardization of features is considered critical to overcome the difficulty in generalizability of radiomics [10], while it is still an open question which factors should be emphasized for improving radiomics robustness.

Dual-energy CT (DECT) is a tremendous innovation in CT technology that allows creation of numerous imaging datasets by enabling discrete acquisitions at more than one energy level [11, 12]. This technology has been coupled with radiomics and yielded as a superior imaging biomarker with encouraging initial results in both oncological and non-oncological fields [13–17]. However, an important prerequisite for widespread application of radiomics on DECT data is a high degree of stability, calling for comprehensive investigation of which factors that influence on DECT radiomics robustness. Difference in single-energy CT (SECT) technique and diverse approaches of DECT acquisition result in CT number variation, and this variation is considered as an important underlying source of radiomics variation [18–21]. Meanwhile, the CT number values also diverge in virtual unenhanced (VUE) images and in virtual monoenergetic images (VMI) across DECT platforms [22, 23]. The energy level of VMI has impact on radiomics robustness [24, 25], and high repeatability of radiomics features could remain stable when the same equivalent energy level was used for VMI generation with different DECT approaches [26]. Accordingly, we hypothesized that the inter-platform variability of radiomic features due to differences in DECT data acquisition and reconstruction may be reduced by creating VMI at appropriate energy levels with comparable CT number values.

In this study, we therefore aimed to evaluate the inter-platform reproducibility of DECT radiomics features in the VUE images and the VMI at energy level of 70 keV (VMI_70keV_) and explore whether variability of CT number value has correlation with the robustness of DECT radiomics features.

## Materials and methods

### Phantom

**Fig. 1:**
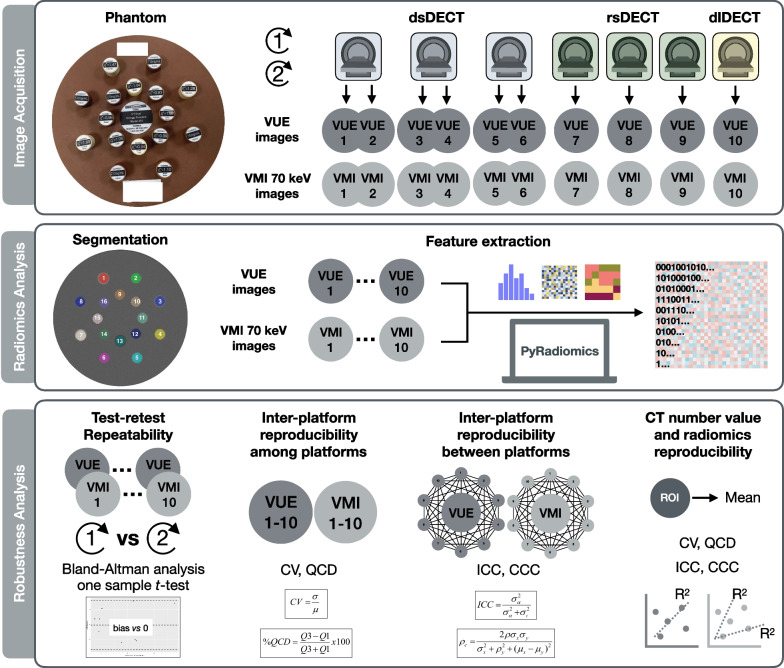
Study workflow. This study was composed of three steps, namely image acquisition, radiomics analysis and robustness analysis. A standardized phantom was scanned on ten platforms on seven DECT-capable scanners of three types with the same voxel and typical abdomen-pelvic examination parameters. Corresponding VUE images and VMI_70keV_ were generated. Pyradiomics was employed to extract 19 first-order and 75 texture radiomics features from ROIs segmented with a rigid registration. The test–retest repeatability was evaluated by Bland–Altman analysis for repeated scans, and the hypothesis that the obtained biases of the radiomics feature values between the scan and rescan was equal to zero was tested by one-sample *t* test. The inter-platform reproducibility among VUE images, and that among VMI_70keV_ images, were assessed by CV and QCD. Inter-platform reproducibility between two particular platform pairs were estimated by ICC and CCC to characterize inter-platform difference across DECT platforms. Since there were ten platforms, forty-five comparisons were performed within the VUE images and within the VMI_70keV_, respectively. CT number and their inter-platform reproducibility were calculated. The correlation between CT number variability of and percentage of robust radiomics features was investigated. dsDECT = dual-source dual-energy CT, rsDECT = rapid kV-switching dual-energy CT, dlDECT = dual-layer dual-energy CT

Figure 1 presents the workflow of this study. The institution’s ethics approval was not required since this was a phantom study. A CT Dual-Energy Phantom Model (Gmamex, Gammex Inc.) was used. This phantom was composed of a 330-mm-in-diameter disk of water-equivalent material and sixteen 28-mm-in-diameter holes for holding interchangeable inserts of various clinical-relevant densities. We selected five iodine inserts with concentrations from 2.0 to 15.0 mg/mL, and eleven rods with densities of 0.44–1.69 g/cm^3^, mimicking wide range of CT number values of human tissues. The inserts were placed to minimize beam-hardening artifacts and kept unchanged across all scans.

### Image acquisition and reconstruction

The phantom was scanned on ten DECT imaging platforms using seven DECT-capable scanners with comparable acquisition and reconstruction parameters (Table 1). Three types of DECT scanners were employed in our study, namely dual-source DECT (dsDECT), rapid kV-switching DECT (rsDECT), and dual-layer detector DECT (dlDECT), to generate images that were comparable to conventional SECT 120-kVp images. Three dsDECT scanners were used, each with two different tube voltage combinations for data acquisition, to provide six DECT imaging platforms. Three rsDECT scanners were used to provide three DECT imaging platforms. One dlDECT scanner at tube voltage of 120 kVp was used to provide the tenth DECT imaging platform. The scan field of view (500 × 500 mm), reconstruction matrix (512 × 512), and slice thickness (5 mm) remained the same for all acquisitions to keep voxel size unchanged. The volume CT dose index, strength of iteration reconstruction algorithm, and reconstruction kernel were chosen to present the typical abdomen-pelvic examinations at our institution. Each scan was repeated several minutes apart with repositioning, to allow test–retest repeatability analysis.

**Table 1:** Dual-energy CT acquisition and reconstruction parameters

No. of platform	Vendor	Scanner	Type	Tube Voltage (kVp)	Milliamperage (mA or mAs)	Rotation Time (sec)	Volume CT dose index (mGy)	Iteration Method	Reconstruction kernel
1	SIEMENS	SOMATOM Drive	dsDECT	80/140	580/224	0.5	20.00	ADMIRE 2	Q40f
2	SIEMENS	SOMATOM Drive	dsDECT	100/140	279/216	0.5	20.04	ADMIRE 2	Q40f
3	SIEMENS	SOMATOM Definition Flash	dsDECT	80/140	531/205	1.0	20.01	SAFIRE 2	Q40s
4	SIEMENS	SOMATOM Definition Flash	dsDECT	100/140	258/199	1.0	19.96	SAFIRE 2	Q40s
5	SIEMENS	SOMATOM Force	dsDECT	70/150	848/212	0.5	20.00	ADMIRE 2	Qr40
6	SIEMENS	SOMATOM Force	dsDECT	100/150	294/147	0.5	20.02	ADMIRE 2	Qr40
7	GE	Discovery CT750 HD	rsDECT	80/140	640^*^	0.6	21.84	ASiR-V 40%	Standard
8	GE	Revolution Apex	rsDECT	80/140	370^*^	1.0	19.75	ASiR-V 40%	Standard
9	GE	Revolution CT	rsDECT	80/140	275^*^	0.8	20.00	ASiR-V 40%	Standard
10	PHILIPS	IQon spectral CT	dlDECT	120	221	0.75	20.00	iDOSE 3	Standard (B)

* represents mA not mAs for GE medical systems. *dsDECT* dual-source dual-energy CT, *rsDECT* rapid kV-switching dual-energy CT, *dlDECT* dual-layer dual-energy CT

Two kinds of images were generated on each DECT imaging platform for radiomic robustness assessment, namely the VUE image and the VMI_70keV_. The VUE images were selected to show the impact of differences in material decomposition techniques between platforms. The VUE images were created using proprietary DECT software tools per vendor-specific material decomposition techniques: Advantage Workstation version 4.7 (GE Healthcare), Syngo.via version VB10 (Siemens Healthineer), and IntelliSpace Portal Workstation version 10 (Philips Healthcare), respectively. The VMI_70keV_ were generated as a gray-scaled, contrast-enhanced benchmark reconstruction relying on comparable linear energy blending approaches on each platform [27–29].

### Segmentation and feature extraction

We applied an open-source ITK-SNAP software version 3.6.0 (http://www.itksnap.org/pmwiki/pmwiki.php) for segmentation, following a rigid registration to minimize variations [30]. Sixteen circular regions-of-interest (ROIs) of 26 pixels (25 mm) in diameter were placed at the center of each insert to present the clinical-relevant densities. To present the true difference among platforms, we did not employ any image preprocessing steps. Python version 3.7.6 (https://www.python.org) with Image Biomarker Standardization Initiative (IBSI)-compliant Pyradiomics package version 3.0 (https://pyradiomics.readthedocs.io/en/latest/) was used to extract the radiomics features from the original images [31]. Since the ROIs were fixed, we excluded the 26 shape-based features. Consequently, 94 radiomics features were extracted from each ROI, namely 19 order features and 75 texture features. The detailed radiomics analysis methods are presented in Additional file 1: Note S1.

### Radiomics robustness analysis

To present the radiomics robustness, the test–retest repeatability and the inter-platform reproducibility were estimated [32]. The test–retest repeatability was assessed using images from repeating scans by Bland–Altman analysis [33]. The percentage of repeatable features was calculated, with a cutoff value of 90% of 16 ROIs [18], indicating the portion of feature scan–rescan measurements that did not exceed the 95% limits of agreement. To test the hypothesis that the obtained biases of the radiomics feature values between the scan and rescan was equal to zero, a one-sample *t* test was performed. The inter-platform reproducibility among the VUE images from ten platforms, and that among the VMI_70keV_ from ten platforms were evaluated, by the coefficient of variation (CV) [34] and the quartile coefficient of dispersion (QCD) [35], respectively, with a cutoff of 10% [30]. To further characterize inter-platform difference across DECT platforms, the inter-platform reproducibility between each platform within the VUE images and within the VMI_70keV_ was estimated to present consistency of two particular platforms, using the intraclass correlation coefficient (ICC) of single rater, absolute agreement, two-way random effects model [36] and the concordance correlation coefficient (CCC) [37, 38], with a cutoff of 0.90 [39, 40]. Since there were ten platforms, forty-five pairs of platforms within the VUE images and within the VMI_70keV_ were compared, respectively, which resulted in ninety comparisons in total. Additional attention was paid to the reproducibility of fourteen individual radiomics features that are important as biomarkers in clinical studies and have been reported to be robust [41–43]. The CT number values and their inter-platform reproducibility were calculated.

### Statistical analysis

The statistical analysis was performed with R language version 4.1.3 (https://www.r-project.org/) within RStudio software version 1.4.1106 (https://www.rstudio.com/). The continuous variables were presented as average ± standard deviation (SD). Proportions of robust features were indicated as percentages. The correlation between inter-platform CT number reproducibility and percentage of radiomics features that met the criteria of reproducibility was quantitatively estimated by Spearman correlation analysis due to the nonnormal distribution of the data. A two-sided *p* value < 0.05 was considered as statistically significant. The detailed statistical analysis methods are presented in Additional file 1: Note S2.

## Results

### Test–retest repeatability analysis of radiomics features

The average percentages ± SD of repeatable radiomics features were 92.02 ± 7.43% and 92.87 ± 4.71% for the VUE images and the VMI_70keV_, respectively, when the cutoff value was 90% of 16 ROIs (Additional file 1: Table S1 and Fig. S1). The biases of the radiomics feature values between the scan and rescan were not significantly different from zero (all *p* > 0.05).

### Inter-platform radiomics reproducibility among all platforms within the VUE images and within the VMI_70keV_

**Fig. 2:**
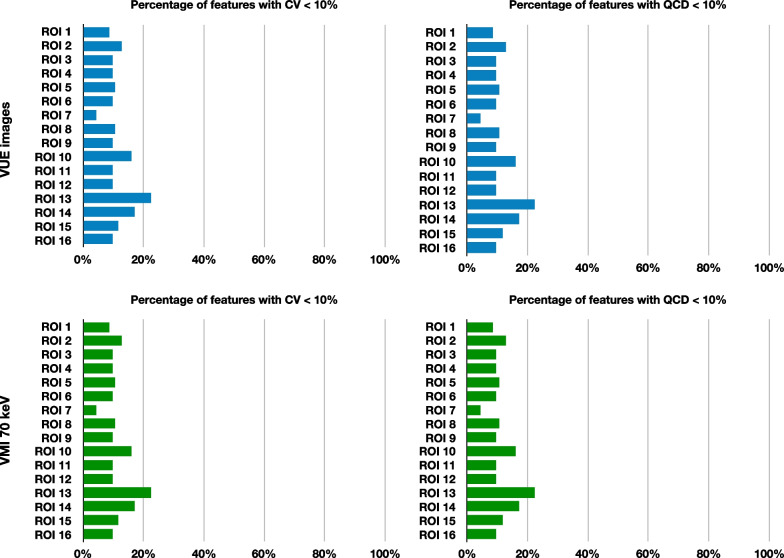
Inter-platform reproducibility of radiomics among all platforms within the VUE images and within the VMI_70keV_. Upper left and right graphs showed percentages of radiomic features that were deemed as inter-platform reproducible among platforms within the VUE images per CV < 10% and QCD < 10%, respectively, according to ROIs. Lower left and right graphs showed percentages of radiomic features that were deemed as inter-platform reproducible among platforms within the VMI_70keV_ per CV < 10% and QCD < 10%, respectively, according to 16 ROIs

**Fig. 3:**
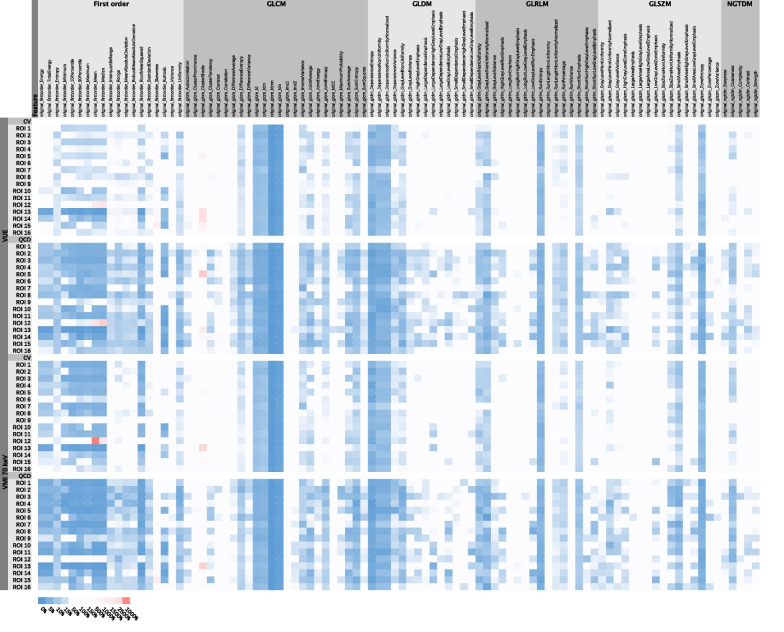
Heatmap of Inter-platform reproducibility of radiomics among all platforms within the VUE images and within the VMI_70keV_. Percentages indicated CV values and QCD values. GLCM = gray-level co-occurrence matrix, GLDM = gray-level dependence matrix, GLRLM = gray-level run-length matrix, GLSZM = gray-level size zone matrix, NGTDM = neighborhood gray-tone difference matrix

The average percentages ± SD of inter-platform reproducible radiomics features were 11.30 ± 4.15% and 28.39 ± 7.19% among all platforms within the VUE images, and 15.16 ± 3.99% and 28.99 ± 13.36% among all platforms within the VMI_70keV_, respectively, when the criteria were CV < 10% and QCD < 10% (Table 2 and Fig. 2). The percentages of radiomics features that met the reproducible criteria ranged from 4.26 to 22.34% for CV < 10% and from 17.02 to 38.30% for QCD < 10% in VUE images, and varied from 9.57 to 20.21% for CV < 10% and from 19.15 to 38.30% for QCD < 10% in VMI_70keV_, according to ROIs (Additional file 1: Table S2). The individual radiomics features showed variable reproducibility (Fig. 3), and the top ten most inter-platform reproducible features among the VUE images and the VMI_70keV_ were mainly the texture features (36 out of 40; Additional file 1: Table S3). The reproducibility of fourteen important radiomics features did not show high reproducibility neither in VUE images (CV values 16.64–579.47%, QCD values 9.11–519.92%) nor in VMI_70keV_ images (CV values 17.61–426.45%, QCD values 9.28–352.37%) (Additional file 1: Table S4).

**Table 2:** Inter-platform reproducibility of radiomics among all platforms within the VUE images and within the VMI_70keV_

Feature class	CV < 10%		CV mean		QCD < 10%		QCD mean	
	VUE (%)	VMI_70keV_ (%)	VUE	VMI_70keV_	VUE (%)	VMI_70keV_ (%)	VUE	VMI_70keV_
First order (19 features)	8.88	26.64	0.5007	0.7358	44.74	50.99	0.4037	0.2484
Texture (75 features)	19.83	20.58	0.4232	0.4042	41.25	38.67	0.2791	0.2625
GLCM (24 features)	16.93	16.15	0.5460	0.4878	30.21	28.91	0.4073	0.3402
GLDM (14 features)	20.54	20.54	0.3081	0.3151	30.36	29.46	0.1839	0.1923
GLRLM (16 features)	7.03	7.03	0.3716	0.3602	25.39	21.48	0.2156	0.2244
GLSZM (16 features)	5.47	8.20	0.3975	0.3966	14.84	15.23	0.2482	0.2558
NGTDM (5 features)	0.00	0.00	0.4024	0.4176	5.00	12.50	0.2328	0.2299
Overall (94 features)	11.30	15.16	0.4388	0.4712	28.39	28.99	0.3043	0.2597

Percentage indicates the percentage of features met the cutoffs for robustness measures (CV < 10% and QCD < 10%). *GLCM* Gray-level co-occurrence matrix, *GLDM* Gray-level dependence matrix, *GLRLM* Gray-level run-length matrix, *GLSZM* Gray-level size zone matrix, *NGTDM* Neighborhood gray-tone difference matrix

### Inter-platform radiomics reproducibility between platform pairs within the VUE images and within the VMI_70keV_

**Fig. 4:**
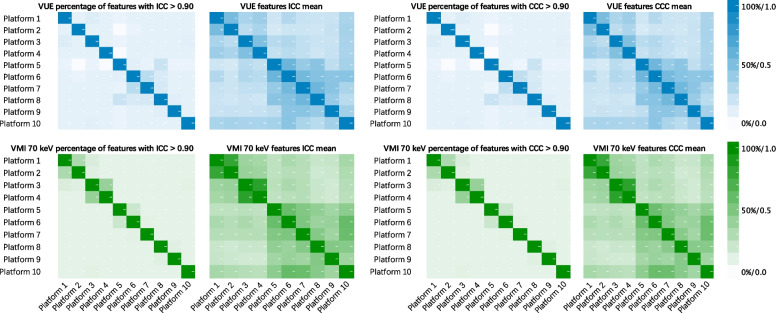
Inter-platform reproducibility of radiomics between platform pairs within the VUE images and within the VMI_70keV_. Upper graphs showed percentages of radiomic features that were deemed as inter-platform reproducible between each platform per ICC > 0.90 and CCC > 0.90, and the mean of ICC and CCC between each platform within the VUE images, respectively, according to 45 comparisons. Lower graphs showed percentages of radiomic features that were deemed as inter-platform reproducible between each platform per ICC > 0.90 and CCC > 0.90, and the mean of ICC and CCC between each platform within the VMI_70keV_, respectively, according to 45 comparisons

The average percentages ± SD of inter-platform reproducible radiomics features were 10.01 ± 3.79% and 9.86 ± 3.67% between each platform within the VUE images, and 11.23 ± 5.78% and 11.23 ± 5.78% within the VMI_70keV_, respectively, when the criteria were ICC > 0.90 and CCC > 0.90 (Table 3 and Fig. 4). The percentages of radiomics features that met the reproducible criteria ranged from 1.06 to 26.60% for ICC > 0.90 and from 1.06 to 24.47% for CCC > 0.90 in VUE images and varied from 9.57 to 40.43%% for ICC > 0.90 and from 9.57 to 41.49% for CCC > 0.90 in VMI_70keV_, according to comparisons (Additional file 1: Table S5 and Fig. S2). The individual radiomics features showed variable reproducibility (Additional file 1: Fig. S3), the top ten most inter-platform reproducible features between each platform within the VUE images and the VMI_70keV_ were mainly the first-order features (36 out of 40; Additional file 1: Table S6). The reproducibility of fourteen important radiomics features did not show high reproducibility neither in VUE images (ICC values, 0.0918–0.4368, CCC values 0.0948–0.4235) nor in VMI_70keV_ images (ICC values 0.0948–0.4469, CCC values 0.0938–0.4345) (Additional file 1: Table S7).

**Table 3:** Inter-platform reproducibility of radiomics between each platform within the VUE images and within the VMI_70keV_

Feature class	ICC > 0.90		ICC mean		CCC > 0.90		CCC mean	
	VUE (%)	VMI_70keV_ (%)	VUE	VMI_70keV_	VUE (%)	VMI_70keV_ (%)	VUE	VMI_70keV_
First order (19 features)	45.03	49.36	0.6654	0.6811	44.68	49.36	0.6584	0.6749
Texture (75 features)	1.13	1.57	0.2324	0.2782	1.04	1.57	0.2247	0.2695
GLCM (24 features)	1.39	2.96	0.2871	0.3146	1.39	2.96	0.2782	0.3050
GLDM (14 features)	1.27	0.95	0.2093	0.2724	1.11	0.95	0.2028	0.2645
GLRLM (16 features)	0.97	0.97	0.2083	0.2644	0.69	0.97	0.2009	0.2560
GLSZM (16 features)	0.69	0.97	0.1963	0.2455	0.69	0.97	0.1895	0.2377
NGTDM (5 features)	1.33	0.44	0.2262	0.2681	1.33	0.44	0.2181	0.2588
Overall (94 features)	10.00	11.23	0.3199	0.3596	9.86	11.23	0.3124	0.3515

Percentage indicates the percentage of features met the cutoffs for robustness measures (ICC > 0.90 and CCC > 0.90). *GLCM* Gray-level co-occurrence matrix, *GLDM* Gray-level dependence matrix, *GLRLM* Gray-level run-length matrix, *GLSZM* Gray-level size zone matrix, *NGTDM* Neighborhood gray-tone difference matrix

### CT number values and radiomics reproducibility

The CT number values varied among platforms within the VUE images and the VMI_70keV_ (Table 4 and Additional file 1: Table S8). The reproducibility of CT number values and percentage of first-order radiomics features that met the criteria of reproducibility showed correlations (Additional file 1: Fig. S4). The negative correlations were found using CV and QCD in both VUE images and VMI_70keV_ (*r*^2^ 0.3870–0.6178, all *p* < 0.001), and positive correlations were estimated using ICC (*r*^2^ = 0.7378, *p* < 0.001) and CCC (*r*^2^ = 0.7717, *p* < 0.001) in the VUE images (Additional file 1: Fig. S4).

**Table 4:** Inter-reproducibility of CT number values

Images	Criteria			
*Inter-platform reproducibility among all platforms within the VUE images and within the VMI*_*70keV*_				
16 ROIs	CV < 10%	CV mean	QCD < 10%	QCD mean
VUE	18.75%	1.1389	81.25%	1.4144
VMI_70keV_	68.75%	6.8006	87.50%	0.3055
*Inter-platform reproducibility between particular platforms within the VUE images and within the VMI*_*70keV*_				
45 Comparisons	ICC > 0.90	ICC mean	CCC > 0.90	CCC mean
VUE	95.56%	0.9803	95.56%	0.9803
VMI_70keV_	100.00%	0.9974	100.00%	0.9972

Percentage indicates the percentage of features met the cutoffs for robustness measures (CV < 10% and QCD < 10%, ICC > 0.90 and CCC > 0.90)

## Discussion

Our study investigated the test–retest repeatability and the inter-platform reproducibility of the VUE images and the VMI_70keV_ in DECT among different platforms, using data from a phantom with inserts of clinical-relevant multiple densities. Our finding demonstrated that the test–retest repeatability of radiomics features derived from different DECT platforms was high, but the inter-platform reproducibility was relatively low, indicating the potential influence of various DECT acquisition and reconstruction techniques. We further characterized the inter-platform difference across DECT platforms by comparing different platform pairs and found that their reproducibility varied according to platforms. The differences in CT number values were deemed to have relation with the inter-platform reproducibility of DECT radiomics features, indicating the potential role of CT number values as an indicator in synchronizing the energy level of VMI of different DECT platforms to improve DECT radiomics robustness.

Our study showed that 11.30% and 28.39% and 15.16% and 28.99% of features were with CV < 10% and QCD < 10%, among the VUE images and the VMI_70keV_ of different DECT platforms, respectively, suggesting the difference in DECT acquisition and reconstruction techniques could be a source of instability. A previous study presented that 17.09% and 27.73% of radiomics features were considered to be reproducible among SECT platforms [18]. This did not support the hypothesis that the differences in DECT data acquisition and reconstruction between platforms may introduce greater variability of radiomic features compared to SECT with a more similar technical set-up [19]. However, in terms of reproducibility, the images acquired via different SECT and DECT platforms, as well as the VUE images and the VMI generated from different DECT platforms, should not be used interchangeably in radiomic studies, even if they were scanned with comparable parameters.

The inter-platform reproducibility between each platform within the VUE images and within the VMI_70keV_ presented varying percentage of radiomics features that met the reproducible criteria. A previous study showed 0.00% and 0.00% of phantom-derived features with CCC > 0.90 in the VUE images and the VMI_65keV_, respectively, between different DECT scanner types, while 2.45–16.15% and 2.71–11.11% of patient-derived features were estimated with CCC > 0.90 in the VUE images and the VMI_65keV_, respectively [19]. The highest percentage of reproducible features were achieved between a third-generation dsDECT scanner and a rsDECT scanner [19]. Another phantom study showed that 66.6–83.5% of radiomics features were with CCC > 0.90 between a third-generation dsDECT scanner and a split-filter DECT scanner within the VMI of the same energy level from 40 to 190 keV [26]. Our study supported that a third-generation dsDECT scanner shared more in common with rsDECT scanners, but did not find similarity between second-generation dsDECT scanners and rsDECT scanners. Indeed, two second-generation dsDECT scanners with two combinations of tube voltages showed high reproducibility. Although the variability among DECT scanners was not greater than that among SECT scanners, the differences in DECT data acquisition and reconstruction between platforms did introduce variability among DECT imaging platforms.

In addition to the overall reproducibility evaluations of radiomics features, we also investigated fourteen individual radiomics features that are currently of interest in clinical research and have been reported to be robust to quantum noise, segmentation variability, and image acquisition [41–43]. However, these radiomics features did not show high reproducibility among DECT platforms, indicating that mitigation of DECT-specific radiomics variability was of importance for generalizability of radiomics models derived from one DECT platform to the other.

The texture features occupied the majority of the top ten most inter-platform reproducible features among the VUE images and among the VMI_70keV_ using CV or QCD as metrics, while the reproducible features between each platform using ICC or CCC were mainly the first-order features. One of the important sources of the inter-platform variability of radiomics is CT number values [18]. The metrics of CV and QCD is considered to present the overall difference among platforms. The outliers of CT number values may have greater impact on the first-order features. Our study found that most of the texture features that survived CV and QCD analysis were related to the homogeneity of the ROI. They were more sensitive to the small noise within ROI than the variations of CT number values. Therefore, the influence of the unstable CT number values on the texture features was less than that on the first-order features. On the other hand, the metrics of ICC and CCC allow evaluation between two specific platforms. The platform five, with obvious differences in CT number values, showed lower reproducibility of the first-order features comparing to other DECT platforms, indicating that the key for improving the reproducibility of first-order features was to keep CT number values stable. In other words, it is possible to improve radiomics reproducibility between DECT platforms by minimizing variability of CT number values, especially the first-order features.

To the best of our knowledge, our study is the first to show the correlation between variability of CT number value and reproducibility of the first-order features derived from DECT data. It is not strange that the first-order features, but not the texture features, were strongly platform-dependent, since the first-order features were more sensitive to difference of CT number values among platforms. It has been considered as a source of difference of radiomics features in SECT that the variability of CT number values across scanners due to the different X-ray spectra of different scanners [20], as well as additional slight differences of the images caused by different calibrations method [30]. CT number values are simple representations of the different imaging appearances, texture features, and quantitative capabilities of DECT images with different technical approaches [11, 12, 22, 23] and may lead to variations among DECT platforms.

CT number values potentially serve as an indicator for improvement for reproducibility among DECT platforms. The lower the variability of CT number values among platforms achieved, the higher the inter-platform reproducibility of the first-order features became. Unlike the VUE images, the VMI could provide an increasing trend of CT number values with decreasing energy level [24]. Meanwhile, the VMI showed lower variability in CT number values than VUE images, when comparable acquisition and reconstruction settings were used [22, 23]. This result might provide insights for reducing the inter-platform difference in DECT radiomic features by better synchronizing energy levels of VMI according to CT number values. It would be more practicable for clinical practice to compare the CT number values, because it is time-consuming to calculate reproducibility of high-dimensional radiomics data extracted from all available energy level of VMIs from different DECT platforms. Future studies should explore the utility of CT number values as an indicator for synchronizing energy level of VMI to improve DECT radiomics robustness.

Additionally, the use of VMI could potentially open more possibilities for radiomics modeling with its flexibility to calculate at low energy level to increase contrast and iodine attenuation or to compute at high energy level to reduce beam-hardening artefacts [44, 45]. The energy level of 70 keV was chosen because this was used as a clinical standard of reference at our institution [18, 46] and has been suggested to be comparable to conventional images [27–29]. However, concerns remained on the potential impact of non-matching energy levels of VMI on radiomics features [24]. Although the choice of synchronized energy level of VMI improved reproducibility between platforms [26], it is still unknown whether the energy level of VMI could alter the underlying minable information. Initial study suggested that VMI at different energy levels could provide varying performance of radiomics models for different clinical tasks [26]. We believe that the choice of energy level of VMI should hence be made to balance radiomics robustness and the specific clinical task.

The implementation of a preprocessing step may be necessary to harmonize data from different platforms using varying DECT techniques. Recently, many preprocessing methods have been introduced into radiomics studies for improving reproducibility of radiomic features, including min–max normalization, z-score normalization, mean normalization, batch effect correction, pixel resampling, Butterworth filtering, ComBat harmonization, radiomics data harmonization models specific to different clinical tasks, etc. [47–54]. As shown in our study, without the preprocessing step, the DECT images are not comparable between platforms in terms of radiomic features. These preprocessing methods have potential to improve the reproducibility of radiomic features among DECT platforms, while their influence on the CT values remains unknown. We believe future studies should test these preprocessing methods to find out which can harmonize data from different platforms using different dual-energy techniques while maintaining CT values.

Our study has limitations that need to be acknowledged. First, we did not investigate the robustness of radiomic features extracted from tumors, but rather from phantom of homogeneous clinical-relevant densities. Our results may not be directly translated to clinical practice, partly due to lacking of texture [18, 46]. However, the phantom allows more specific results in humans benefiting by its similarity to human density [55]. Second, we did not identify at which energy levels of VMIs to accomplish the highest inter-platform reproducibility. Nevertheless, our findings showed the possibility of harmonizing inter-platform radiomics features by synchronizing energy levels of VMIs and showed the potential role of CT number values in guiding selection of energy levels for this purpose. With multiple phantom scans on different platforms, one may be able to adjust energy levels of different imaging platforms to obtain similar CT number values for the same object. Therefore, a pre-calibrated lookup table may be possible to account for the differences of data acquisition and image reconstruction from different DECT platforms to improve DECT-derived radiomics robustness. Third, we only investigated fourteen individual radiomics features in detail. These radiomics features were considered to be clinically important, but the ability of radiomics features for clinical interpretation or classification varied according to specific tasks. Therefore, further studies with patient images on specific clinical applications are warranted. Last, the results of our study should be carefully interpreted as hypothesis generating. We neither perform experiments to test the feasibility of CT numbers as a correction factor for reducing inter-platform variability nor conduct experiments to investigate the potential impact of a preprocessing step on the reproducibility of radiomics features. Our findings may provide insights on improvement of the inter-platform reproducibility, and our ongoing work is verifying the hypothesis.

To conclude, we have demonstrated that the radiomics features extracted from the VUE images and the VMI_70keV_ are not highly reproducible across different DECT platforms, despite using comparable acquisition and reconstruction parameters. DECT-derived radiomic models must be interpreted with caution due to the doubtful generalizability. The variability of CT number values is correlated with the reproducibility of the first-order features in radiomics, implying a potential way to mitigate radiomics instability among DECT platforms. Future studies should investigate the possibility of synchronizing energy levels of VMI among different DECT platforms with an appropriate preprocessing step to improve the robustness of DECT-derived radiomics.

## Supplementary Information

**Additional file 1.** Supplementay Materials.

## Data Availability

All data generated or analyzed during this study are included in this published article and its supplementary information files.

## Abbreviations

CCC: Concordance correlation coefficient
CV: Coefficient of variation
DECT: Dual-energy computed tomography
dlDECT: Dual-layer detector dual-energy computed tomography
dsDECT: Dual-source dual-energy computed tomography
ICC: Intraclass correlation coefficient
QCD: Quartile coefficient of dispersion
ROI: Region of interest
rsDECT: Rapid kV-switching dual-energy computed tomography
SD: Standard deviation
SECT: Single-energy computed tomography
VMI: Virtual monoenergetic images
VUE: Virtual unenhanced images
