# Supplementary material for: Robustness of radiomics features of virtual unenhanced and virtual monoenergetic images in dual-energy CT among different imaging platforms and potential role of CT number variability
Source: Insights Imaging. 2023 May 11;14:79. doi: 10.1186/s13244-023-01426-5 (PMC10175529; doi:10.1186/s13244-023-01426-5)

ELECTRONIC SUPPLEMENTARY MATERIAL

Robustness of radiomics features of virtual unenhanced and virtual monoenergetic images in dual-energy CT among different imaging platforms and potential role of CT number variability

List of Supplementary Materials:

Supplementary Note

Supplementary Note S1. Radiomics analysis method.

Supplementary Note S2. Statistical analysis method.

Supplementary Tables

Supplementary Table S1. Test-retest repeatability radiomics between scan and re-scan.

Supplementary Table S2. Inter-platform reproducibility of radiomics among all platforms within the VUE images and within the VMI_{70keV}.

Supplementary Table S3. Ten most inter-platform reproducible radiomic features among all platforms within the VUE and within the VMI_{70keV}.

Supplementary Table S4. Inter-platform reproducibility of fourteen important radiomics features among all platforms within the VUE images and within the VMI_{70keV}.

Supplementary Table S5. Inter-platform reproducibility of radiomics between each platform within the VUE images and the VMI_{70keV}.

Supplementary Table S6. Ten most inter-platform reproducible radiomics between each platform within the VUE images and the VMI_{70keV}.

Supplementary Table S7. Inter-platform reproducibility of fourteen important radiomics features between each platform within the VUE images and the VMI_{70keV}.

Supplementary Table S8. CT number values per ROI of each platform.

Supplementary Figure

Supplementary Figure S1. Heatmap of Test-retest repeatability.

Supplementary Figure S2. Inter-platform reproducibility of radiomics between each platform within the VUE images and the VMI_{70keV}.

Supplementary Figure S3. Heatmap of inter-platform reproducibility of radiomics between each platform within the VUE images and the VMI_{70keV}.

Supplementary Figure S4. Correlation of inter-platform CT number reproducibility and radiomics reproducibility.

Supplementary Note S1. Radiomics analysis method.

(a) ROI segmentation

We drew the ROIs by using an open-source software, ITK-SNAP software version 3.6.0 (http://www.itksnap.org/pmwiki/pmwiki.php), following a rigid registration to minimize variations [1,2]. We copied the ROIs from one examination to another. Sixteen circular regions-of-interest (ROI 1 to 16) were circles of 25 mm (26 pixels) in diameter set at the center of each rod, to cover each rod as much as possible and avoid touching its edge.

The following illustrates the placing of 16 ROIs for corresponding inserts. Sixteen inserts were used to present multiple clinical-relevant densities, including five rods with iodine (Iod) concentrations of 2.0 mg/mL, 2.5 mg/mL, 5.0 mg/mL, 7.5 mg/mL, and 15.0 mg/mL, and eleven rods with human body densities, namely lung (0.44 g/cm^3), adipose (0.93 g/cm^3), breast (0.96 g/cm^3), solid water (0.99 g/cm^3), brain (1.04 g/ cm^3), liver (1.06 g/cm^3), inner bone (1.09 g/cm^3), bone (1.10 g/cm^3), cortical bone (CB) 2-30% (1.28 g/cm^3), cortical bone (CB) 2-50% (1.47 g/cm^3), and cortical bone (1.69 g/cm^3).

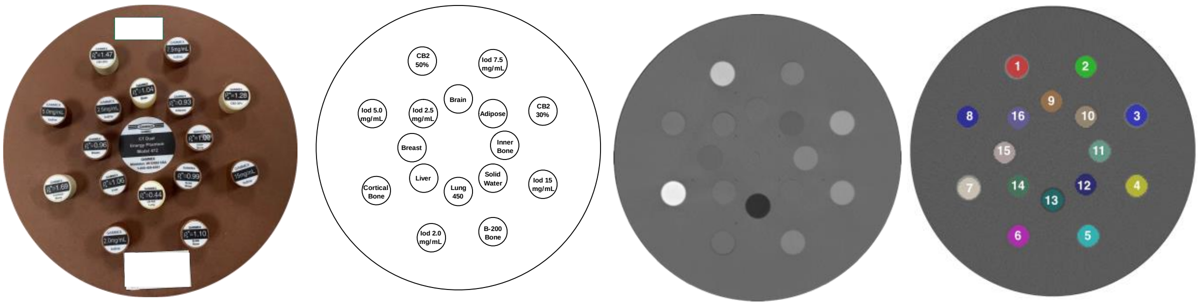

(b) Feature extraction

To present the true difference among platforms, we did not employ any pre-processing image procedure. Python version 3.7.6 (https://www.python.org) with Image Biomarker Standardisation Initiative (IBSI)-compliant Pyradiomics package version 3.0 (https://pyradiomics.readthedocs.io/en/latest/) was used to extract the radiomics features from the original images. Since the ROIs were stable, we excluded 26 shape-based features. Consequently, 94 radiomics features were extracted from each ROI, namely 19 order features and 75 texture features.

(c) CT number values

The CT number value was directly extracted from the radiomics analysis. One of the extracted radiomics features (fistorder_Mean) calculated the mean HU values of the ROI. This value was considered as the CT number value. We have confirmed the data with manual measurement using imQuest software version 7.1 (https://deckard.duhs.duke.edu/~samei/tg233.html) [3, 4]. The CT number values were approximately the same between these two methods. We computed the CT number values within 3 consecutive axial slices from 2 repeated scans. In total, there were 3 × 2 = 6 ROIs for per insert on each platform.

References:

1. Berenguer R, Pastor-Juan MDR, Canales-Vázquez J et al (2018) Radiomics of CT features may be nonreproducible and redundant: influence of CT acquisition parameters. Radiology 288(2):407-415
2. Chen Y, Zhong J, Wang L et al (2022) Robustness of CT radiomics features: consistency within and between single-energy CT and dual-energy CT. Eur Radiol. doi: 10.1007/s00330-022-08628-3
3. Samei E, Richard S (2015) Assessment of the dose reduction potential of a model-based iterative reconstruction algorithm using a task-based performance metrology. Med Phys 42(1):314-323
4. Samei E, Bakalyar D, Boedeker KL et al (2019) Performance evaluation of computed tomography systems: Summary of AAPM Task Group 233. Med Phys 46(11):e735-e756

Supplementary Note S2. Statistical analysis method.

The statistical analysis was performed with R language version 4.1.3 (https://www.r-project.org/) within RStudio version 1.4.1106 (https://www.rstudio.com/) using DescTools package version 0.99.44 and BlandAltmanLeh package version 0.3.1. Radionics robustness analyses were performed. Comparison of continuous variables were presented as average ± standard deviation (SD). Proportions of robust features were indicated as percentages. A two-sided p value < 0.05 was considered statistically significant.

(a) Test-retest repeatability analysis of radiomics features

For test-retest analysis, all repeated scans were involved. Radiomics features were extracted on 16 ROIs of the most representative middle three layers of images from two repeating scans with the same acquisition parameters on the same scanner. The ROIs were copied from the first scan to the second to preclude ROI variations. The repeatability was assessed by Bland-Altman analysis [1, 2]. The percentage of repeatable features were calculated with cutoff values of 85%, 90%, and 95% of 16 ROIs. To simplify the analysis, the cutoff value of 90% was selected as the main effect. To test the hypothesis that the obtained biases of the radiomics feature values between the scan and re-scan were equal to zero, a one-sample *t*-test was performed.

(b) Inter-platform reproducibility analysis of radiomics features among all platforms within all VUE images and within the VMI_{70keV}

The inter-platform reproducibility analysis was performed among all VUE images from ten platforms, and among all VMI_{70keV} from ten platforms, using the coefficient of variation (CV) [3] and quartile coefficient of dispersion (QCD) [4] with cutoff values of 5%, 10%, and 15% were used as measures to evaluate the reproducibility. To simplify the analysis, the cutoff value of 10% was selected as the main effect.

The following illustrated the formulae used in our study, which followed Berenguer et al [5] and Chen et al [6].

CV formula:

$$CV = \frac{\sigma}{\mu}$$

CV is defined as the ratio of the standard deviation (σ) to the mean (μ) [3].

QCD formula:

$$\%QCD = \frac{Q3 - Q1}{Q3 + Q1} x100$$

where Q1 and Q3 are the first and third quartiles, respectively [4].

(d) Inter-platform reproducibility analysis of radiomics features between each platform within all VUE and VMI_{70keV} images

The inter-platform reproducibility between each platform within the VUE images and within the VMI_{70keV} estimated the consistency of two particular platforms within the VUE images and within the VMI_{70keV}, respectively. Since there were ten platforms included, forty-five pairs of platforms in the VUE images and the VMI_{70keV} each were compared (9 × 10 / 2 = 45 pairs for each type of images). Consequently, 90 comparisons were performed in total (45 × 2 = 90). The ICC using single rater, absolute agreement, two-way random effects model [7], and the CCC [8, 9] and was employed as comparative measures with cutoff values of 0.85, 0.90, and 0.95. To simplify the analysis, the cutoff value of 0.90 was selected as the main effect. The formulae used in our study followed Berenguer et al [5] and Chen et al [6].

The following illustrated the formulae used in our study, which followed Berenguer et al [5] and Chen et al [6].

ICC formula:

$$ICC = \frac{\sigma_\alpha^2}{\sigma_\alpha^2 + \sigma_\epsilon^2}$$

where σ^2_α is the pooled variance within the subjects, and σ2ε is the trait between subjects [7].

CCC formula:

$$\rho_c = \frac{2\rho\sigma_x\sigma_y}{\sigma_x^2 + \rho_y^2 + (\mu_x - \mu_y)^2}$$

where μ_x and μ_y are the means for the 2 variables and σ^2_x and σ^2_y are the corresponding variances. Ρ is the correlation coefficient between the two variables [8, 9].

(e) Inter-platform reproducibility of CT number values

In our study, the CT number value was treated as a radiomics feature. Therefore, the calculation of inter-platform reproducibility of CT number values followed the same method for radiomics features.

(f) Correlation analysis

The correlation between CT number reproducibility and that of radiomics features was quantitatively estimated by Spearman correlation analysis due to the nonnormal distribution of data. The following correlations were investigated:

(1) inter-platform CV of CT number values × percentage features with CV < 10%
(2) inter-platform QCD of CT number values × percentage features with QCD < 10%
(3) inter-platform ICC of CT number values × percentage of with ICC > 0.90
(4) inter-platform CCC of CT number values × percentage of with QCD > 0.90
each of these correlations was investigated according to overall, first order, and texture features, in the VUE images and the VMI_{70keV}.

Therefore, there were 4 × 3 × 2 = 24 correlations investigated in total. The correlation was estimated using Spearman correlation analysis due to nonnormal distribution of data.

(g) Considerations on cutoff values

The radiomics community so far did not come to a consensus on which metric and what cutoff is the most adequate to use in a radiomics setting [10,11]. The cutoffs of ICC range from 0.5 to 0.85, and cutoffs of CCC range from 0.7 to 0.85. The cutoff of CV was recommended to be < 15% by the European Society of Radiology [12]. The cutoffs of CV and QCD were set at 0.85, 0.90 and 0.95 in a representative study [3].

We used the cutoff of 0.90 as the primary outcome of our study. As Koo and Li suggested, the cutoffs of ICC were: poor, less than 0.50; moderate, 0.50 to 0.75; good 0.75 to 0.90; excellent, greater than 0.90 [7]. Since there is no consensus reached on what cutoff is the most adequate to use in radiomics robustness research, we provided the results using cutoffs of 0.85 and 0.95 as Supplementary materials, to allow a more complete assessment.

References:

1. Sullivan DC, Obuchowski NA, Kessler LG et al (2015) RSNA-QIBA Metrology Working Group. Metrology standards for quantitative imaging biomarkers. Radiology 277(3):813–825
2. Bland JM, Altman DG (1999) Measuring agreement in method comparison studies. Stat Methods Med Res 8(2):135–160
3. Reed GF, Lynn F, Meade BD (2002) Use of coefficient of variation in assessing variability of quantitative assays. Clin Diagn Lab Immunol 9(6):1235–1239
4. Bonett DG (2006) Confidence interval for a coefficient of quartile variation. Comput Stat Data Anal 50(11):2953–2957
5. Berenguer R, Pastor-Juan MDR, Canales-Vázquez J et al (2018) Radiomics of CT features may be nonreproducible and redundant: influence of CT acquisition parameters. Radiology 288(2):407-415
6. Chen Y, Zhong J, Wang L et al (2022) Robustness of CT radiomics features: consistency within and between single-energy CT and dual-energy CT. Eur Radiol. doi: 10.1007/s00330-022-08628-3
7. Koo TK, Li MY (2016) A Guideline of Selecting and Reporting Intraclass Correlation Coefficients for Reliability Research. J Chiropr Med 15(2):155–163
8. Lin LI (1989) A concordance correlation coefficient to evaluate reproducibility. Biometrics 45(1):255–268
9. Lin LI (2000) A note on the concordance correlation coefficient. Biometrics 56(1):324–325
10. Traverso A, Wee L, Dekker A, Gillies R (2018) Repeatability and Reproducibility of Radiomic Features: A Systematic

Review. Int J Radiat Oncol Biol Phys 102(4):1143-1158

11. Pfaehler E, Zhovannik I, Wei L et al (2021) A systematic review and quality of reporting checklist for repeatability and reproducibility of radiomic features. Phys Imaging Radiat Oncol 20:69-75
12. European Society of Radiology (ESR). (2020) ESR Statement on the Validation of Imaging Biomarkers. Insights Imaging 11(1):76

Supplementary Table S1. Test-retest repeatability radiomics between scan and re-scan.

Platform	VUE images			VMI_{70keV}		
	B-A > 0.85	B-A > 0.90	B-A > 0.95	B-A > 0.85	B-A > 0.90	B-A > 0.95
Platform 1	94.68%	82.98%	42.55%	100.00%	95.74%	55.32%
Platform 2	100.00%	75.53%	36.17%	100.00%	96.81%	54.26%
Platform 3	100.00%	97.87%	73.40%	100.00%	89.36%	60.64%
Platform 4	100.00%	91.49%	43.62%	100.00%	97.87%	57.45%
Platform 5	100.00%	95.74%	53.19%	100.00%	93.62%	55.32%
Platform 6	100.00%	92.55%	39.36%	100.00%	85.11%	40.43%
Platform 7	100.00%	96.81%	56.38%	100.00%	95.74%	43.62%
Platform 8	100.00%	100.00%	43.62%	100.00%	97.87%	24.47%
Platform 9	100.00%	92.55%	55.32%	100.00%	87.23%	35.11%
Platform 10	100.00%	94.68%	34.04%	100.00%	89.36%	28.72%
Overall	99.47%	92.02%	47.77%	100.00%	92.87%	45.53%

Note: percentages indicated the portion of feature scan-rescan measurements that did not exceed the 95% limits of agreement (LoA).

Supplementary Table S2. Inter-platform reproducibility of radiomics among all platforms within the VUE images and within the VMI_{70keV}.

ROI	CV<15%	CV<10%	CV<5%	CV mean	QCD<15%	QCD<10%	QCD<5%	QCD mean
VUE images								
ROI 1	21.28%	8.51%	2.13%	0.4300	41.49%	21.28%	11.70%	0.2472
ROI 2	25.53%	12.77%	3.19%	0.2752	60.64%	38.30%	15.96%	0.1657
ROI 3	22.34%	9.57%	2.13%	0.3354	47.87%	34.04%	15.96%	0.1916
ROI 4	21.28%	9.57%	3.19%	0.3094	58.51%	38.30%	8.51%	0.1793
ROI 5	25.53%	10.64%	3.19%	0.3962	42.55%	29.79%	11.70%	0.6981
ROI 6	18.09%	9.57%	2.13%	0.3716	43.62%	27.66%	9.57%	0.2306
ROI 7	14.89%	4.26%	2.13%	0.6213	30.85%	21.28%	4.26%	0.3540
ROI 8	19.15%	10.64%	2.13%	0.2859	51.06%	34.04%	11.70%	0.1693
ROI 9	12.77%	9.57%	2.13%	0.3865	38.30%	19.15%	7.45%	0.2433
ROI 10	20.21%	15.96%	2.13%	0.3997	37.23%	20.21%	9.57%	0.2979
ROI 11	23.40%	9.57%	3.19%	0.3116	47.87%	28.72%	10.64%	0.1890
ROI 12	18.09%	9.57%	4.26%	0.7775	44.68%	23.40%	7.45%	0.8327
ROI 13	26.60%	22.34%	11.70%	0.5834	53.19%	32.98%	14.89%	0.3804
ROI 14	26.60%	17.02%	3.19%	0.5890	57.45%	37.23%	14.89%	0.2323
ROI 15	21.28%	11.70%	3.19%	0.5704	50.00%	30.85%	11.70%	0.2233
ROI 16	13.83%	9.57%	2.13%	0.3779	41.49%	17.02%	4.26%	0.2342
Overall	20.68%	11.30%	3.26%	0.4388	46.68%	28.39%	10.64%	0.3043
VMI_{70keV} images								
ROI 1	25.53%	20.21%	8.51%	0.3302	42.55%	26.60%	17.02%	0.2146
ROI 2	31.91%	18.09%	3.19%	0.3397	50.00%	38.30%	19.15%	0.1832
ROI 3	27.66%	19.15%	9.57%	0.3009	57.45%	38.30%	18.09%	0.1842
ROI 4	30.85%	17.02%	3.19%	0.3535	41.49%	35.11%	17.02%	0.2227
ROI 5	27.66%	18.09%	5.32%	0.3488	51.06%	31.91%	19.15%	0.2042
ROI 6	18.09%	9.57%	3.19%	0.3584	43.62%	32.98%	12.77%	0.2501
ROI 7	23.40%	18.09%	6.38%	0.5588	38.30%	24.47%	14.89%	0.2730
ROI 8	22.34%	15.96%	3.19%	0.3128	53.19%	37.23%	11.70%	0.1688
ROI 9	14.89%	9.57%	2.13%	0.4243	46.81%	24.47%	4.26%	0.2400
ROI 10	23.40%	17.02%	3.19%	0.3683	36.17%	23.40%	10.64%	0.2505
ROI 11	29.79%	18.09%	3.19%	0.3253	54.26%	31.91%	17.02%	0.1978
ROI 12	18.09%	9.57%	3.19%	1.6997	34.04%	20.21%	5.32%	0.3009
ROI 13	24.47%	18.09%	11.70%	0.6872	42.55%	24.47%	18.09%	0.6342
ROI 14	22.34%	13.83%	3.19%	0.3713	44.68%	24.47%	12.77%	0.2331
ROI 15	24.47%	10.64%	3.19%	0.3895	52.13%	30.85%	10.64%	0.3681
ROI 16	14.89%	9.57%	2.13%	0.3711	32.98%	19.15%	5.32%	0.2291
Overall	23.74%	15.16%	4.65%	0.4712	45.08%	28.99%	13.36%	0.2597

Note: percentage indicates the portion of features that met the criteria.

Supplementary Table S3. Ten most inter-platform reproducible radiomic features among all platforms within the VUE images and within the VMI_{70keV}.

Feature name	CV	Feature name	QCD
VUE images			
glcm_Idmn	0.68%	glcm_Idmn	0.51%
glcm_Idn	2.00%	glcm_Idn	1.48%
gldm_DependenceEntropy	5.99%	gldm_DependenceEntropy	3.15%
glrlm_RunEntropy	7.29%	glcm_Id	4.64%
glcm_Id	7.58%	glszm_ZoneEntropy	4.68%
gldm_DependenceNonUniformity	7.81%	gldm_DependenceNonUniformity	4.85%
gldm_DependenceNonUniformityNormalized	7.81%	gldm_DependenceNonUniformityNormalized	4.85%
glcm_Idm	8.34%	glcm_Idm	4.98%
glszm_ZoneEntropy	8.41%	glrlm_RunEntropy	5.48%
glrlm_GrayLevelNonUniformityNormalized	12.74%	firstorder_RootMeanSquared	6.63%
VMI_{70keV} images			
glcm_Idmn	0.70%	glcm_Idmn	0.53%
glcm_Idn	2.17%	glcm_Idn	1.76%
gldm_DependenceEntropy	5.21%	gldm_DependenceEntropy	2.93%
glrlm_RunEntropy	6.62%	firstorder_RootMeanSquared	4.34%
glszm_ZoneEntropy	6.79%	glszm_ZoneEntropy	4.49%
gldm_DependenceNonUniformity	8.17%	glrlm_RunEntropy	5.11%
gldm_DependenceNonUniformityNormalized	8.17%	firstorder_90Percentile	5.16%
glcm_Id	8.57%	glcm_Id	5.80%
glcm_Idm	9.65%	gldm_DependenceNonUniformity	5.81%
firstorder_RootMeanSquared	10.10%	gldm_DependenceNonUniformityNormalized	5.81%

Supplementary Table S4. Inter-platform reproducibility of fourteen important radiomics features among all platforms within the VUE images and within the VMI_{70keV}.

No.	Feature name	Feature family	CV in VUE images	QCD in VUE images	CV in VMI_{70keV} images	QCD in VMI_{70keV} images
3	Entropy	First order	18.81%	10.94%	18.69%	9.87%
19	Uniformity	First order	16.64%	9.11%	17.61%	9.28%
22	Cluster Shade	GLCM	579.47%	519.92%	426.45%	352.37%
40	Maximum Probability	GLCM	30.89%	19.62%	32.38%	20.17%
52	Large Dependence High Gray Level Emphasis	GLDM	42.34%	26.64%	49.35%	27.60%
63	Long Run High Gray Level Emphasis	GLRLM	65.12%	36.79%	70.24%	41.40%
64	Long Run Low Gray Level Emphasis	GLRLM	74.33%	43.62%	61.53%	40.32%
65	Low Gray Level Run Emphasis	GLRLM	39.29%	21.54%	31.66%	20.05%
72	Short Run High Gray Level Emphasis	GLRLM	45.35%	24.46%	47.13%	25.68%
73	Short Run Low Gray Level Emphasis	GLRLM	36.19%	20.86%	29.65%	18.02%
90	Busyness	NGTDM	46.77%	26.63%	40.59%	23.56%
91	Coarseness	NGTDM	21.95%	12.41%	18.27%	9.78%
93	Contrast	NGTDM	28.26%	18.92%	31.93%	22.82%
94	Strength	NGTDM	47.78%	27.17%	56.13%	27.70%

Note: No. indicates the number of features in the Pyradiomics. Presents as average of CV valves and average of QCD values of 16 ROIs. GLCM = gray-level co-occurrence matrix, GLDM = gray-level dependence matrix, GLRLM = gray-level run-length matrix, GLSZM = gray-level size zone matrix, NGTDM = neighborhood gray-tone difference matrix.

Supplementary Table S5. Inter-platform reproducibility of radiomics between each platform within the VUE images and the VMI_{70keV}.

Comparison	ICC > 0.85	ICC > 0.90	ICC > 0.95	ICC mean	CCC > 0.85	CCC > 0.90	CCC > 0.95	CCC mean
VUE images								
Platform 1 vs 2	11.70%	9.57%	9.57%	0.5310	10.64%	9.57%	9.57%	0.5203
Platform 1 vs 3	14.89%	12.77%	11.70%	0.3275	14.89%	12.77%	11.70%	0.3203
Platform 1 vs 4	9.57%	9.57%	9.57%	0.3808	9.57%	9.57%	9.57%	0.3705
Platform 1 vs 5	7.45%	7.45%	1.06%	0.1836	7.45%	7.45%	1.06%	0.1788
Platform 1 vs 6	9.57%	9.57%	9.57%	0.2927	9.57%	9.57%	9.57%	0.2858
Platform 1 vs 7	9.57%	9.57%	9.57%	0.2115	9.57%	9.57%	9.57%	0.2075
Platform 1 vs 8	9.57%	9.57%	9.57%	0.1945	9.57%	9.57%	9.57%	0.1908
Platform 1 vs 9	9.57%	9.57%	9.57%	0.2047	9.57%	9.57%	9.57%	0.2013
Platform 1 vs 10	9.57%	9.57%	9.57%	0.3297	9.57%	9.57%	9.57%	0.3231
Platform 2 vs 3	9.57%	9.57%	9.57%	0.2600	9.57%	9.57%	9.57%	0.2538
Platform 2 vs 4	10.64%	9.57%	9.57%	0.3265	10.64%	9.57%	9.57%	0.3177
Platform 2 vs 5	6.38%	1.06%	0.00%	0.1566	6.38%	1.06%	0.00%	0.1516
Platform 2 vs 6	9.57%	7.45%	7.45%	0.1918	9.57%	7.45%	7.45%	0.1875
Platform 2 vs 7	9.57%	9.57%	9.57%	0.1919	9.57%	9.57%	9.57%	0.1879
Platform 2 vs 8	9.57%	9.57%	7.45%	0.1792	9.57%	9.57%	7.45%	0.1753
Platform 2 vs 9	9.57%	9.57%	7.45%	0.2152	9.57%	9.57%	7.45%	0.2098
Platform 2 vs 10	9.57%	9.57%	9.57%	0.3443	9.57%	9.57%	9.57%	0.3344
Platform 3 vs 4	9.57%	9.57%	9.57%	0.4977	9.57%	9.57%	9.57%	0.4847
Platform 3 vs 5	7.45%	7.45%	1.06%	0.2747	7.45%	7.45%	1.06%	0.2660
Platform 3 vs 6	9.57%	9.57%	9.57%	0.3029	9.57%	9.57%	9.57%	0.2954
Platform 3 vs 7	9.57%	9.57%	9.57%	0.2405	9.57%	9.57%	9.57%	0.2351
Platform 3 vs 8	9.57%	9.57%	9.57%	0.2042	9.57%	9.57%	9.57%	0.1999
Platform 3 vs 9	9.57%	9.57%	9.57%	0.2029	9.57%	9.57%	9.57%	0.1980
Platform 3 vs 10	11.70%	10.64%	9.57%	0.3355	11.70%	10.64%	9.57%	0.3274
Platform 4 vs 5	7.45%	2.13%	0.00%	0.2262	7.45%	1.06%	0.00%	0.2184
Platform 4 vs 6	9.57%	9.57%	7.45%	0.2685	9.57%	9.57%	7.45%	0.2603
Platform 4 vs 7	9.57%	9.57%	9.57%	0.2389	9.57%	9.57%	9.57%	0.2328
Platform 4 vs 8	9.57%	9.57%	7.45%	0.1829	9.57%	9.57%	7.45%	0.1788
Platform 4 vs 9	9.57%	9.57%	7.45%	0.2158	9.57%	9.57%	7.45%	0.2097
Platform 4 vs 10	9.57%	9.57%	9.57%	0.3280	9.57%	9.57%	9.57%	0.3192
Platform 5 vs 6	12.77%	10.64%	7.45%	0.5778	12.77%	9.57%	5.32%	0.5649
Platform 5 vs 7	8.51%	8.51%	1.06%	0.4086	8.51%	6.38%	1.06%	0.3989
Platform 5 vs 8	22.34%	20.21%	10.64%	0.4540	22.34%	20.21%	8.51%	0.4463
Platform 5 vs 9	9.57%	7.45%	4.26%	0.2497	7.45%	7.45%	4.26%	0.2422
Platform 5 vs 10	7.45%	7.45%	1.06%	0.3455	7.45%	7.45%	1.06%	0.3346
Platform 6 vs 7	31.91%	26.60%	17.02%	0.5478	30.85%	24.47%	17.02%	0.5396
Platform 6 vs 8	15.96%	14.89%	11.70%	0.4734	15.96%	14.89%	11.70%	0.4648
Platform 6 vs 9	9.57%	9.57%	9.57%	0.4506	9.57%	9.57%	9.57%	0.4385
Platform 6 vs 10	9.57%	9.57%	9.57%	0.4576	9.57%	9.57%	9.57%	0.4474
Platform 7 vs 8	17.02%	15.96%	11.70%	0.5674	15.96%	15.96%	11.70%	0.5561
Platform 7 vs 9	9.57%	9.57%	9.57%	0.4564	9.57%	9.57%	9.57%	0.4456
Platform 7 vs 10	10.64%	9.57%	9.57%	0.3298	10.64%	9.57%	9.57%	0.3230
Platform 8 vs 9	12.77%	11.70%	9.57%	0.4054	12.77%	11.70%	9.57%	0.3965
Platform 8 vs 10	10.64%	9.57%	9.57%	0.2700	10.64%	9.57%	9.57%	0.2645
Platform 9 vs 10	9.57%	9.57%	9.57%	0.3603	9.57%	9.57%	9.57%	0.3515

Overall	10.80%	10.00%	8.27%	0.3199	10.69%	9.86%	8.18%	0.3124
VMI_{70keV} images								
Platform 1 vs 2	47.87%	31.91%	17.02%	0.7916	41.49%	31.91%	17.02%	0.7825
Platform 1 vs 3	14.89%	13.83%	11.70%	0.3839	14.89%	13.83%	11.70%	0.3747
Platform 1 vs 4	10.64%	9.57%	9.57%	0.3479	9.57%	9.57%	9.57%	0.3384
Platform 1 vs 5	9.57%	9.57%	9.57%	0.2491	9.57%	9.57%	9.57%	0.2421
Platform 1 vs 6	9.57%	9.57%	9.57%	0.3266	9.57%	9.57%	9.57%	0.3177
Platform 1 vs 7	9.57%	9.57%	9.57%	0.2834	9.57%	9.57%	9.57%	0.2771
Platform 1 vs 8	10.64%	9.57%	9.57%	0.2093	9.57%	9.57%	9.57%	0.2052
Platform 1 vs 9	9.57%	9.57%	9.57%	0.2171	9.57%	9.57%	9.57%	0.2120
Platform 1 vs 10	9.57%	9.57%	9.57%	0.3462	9.57%	9.57%	9.57%	0.3376
Platform 2 vs 3	13.83%	11.70%	9.57%	0.3964	12.77%	11.70%	9.57%	0.3864
Platform 2 vs 4	9.57%	9.57%	9.57%	0.3705	9.57%	9.57%	9.57%	0.3604
Platform 2 vs 5	9.57%	9.57%	9.57%	0.2492	9.57%	9.57%	9.57%	0.2419
Platform 2 vs 6	9.57%	9.57%	9.57%	0.2820	9.57%	9.57%	9.57%	0.2746
Platform 2 vs 7	9.57%	9.57%	9.57%	0.2770	9.57%	9.57%	9.57%	0.2707
Platform 2 vs 8	9.57%	9.57%	9.57%	0.1829	9.57%	9.57%	9.57%	0.1800
Platform 2 vs 9	9.57%	9.57%	9.57%	0.1948	9.57%	9.57%	9.57%	0.1903
Platform 2 vs 10	9.57%	9.57%	9.57%	0.3079	9.57%	9.57%	9.57%	0.3007
Platform 3 vs 4	62.77%	40.43%	11.70%	0.8413	58.51%	40.43%	10.64%	0.8338
Platform 3 vs 5	9.57%	9.57%	9.57%	0.2682	9.57%	9.57%	9.57%	0.2606
Platform 3 vs 6	9.57%	9.57%	9.57%	0.3231	9.57%	9.57%	9.57%	0.3149
Platform 3 vs 7	10.64%	9.57%	9.57%	0.2839	10.64%	9.57%	9.57%	0.2771
Platform 3 vs 8	9.57%	9.57%	9.57%	0.2070	9.57%	9.57%	9.57%	0.2025
Platform 3 vs 9	9.57%	9.57%	9.57%	0.1758	9.57%	9.57%	9.57%	0.1718
Platform 3 vs 10	10.64%	10.64%	10.64%	0.2848	10.64%	10.64%	10.64%	0.2777
Platform 4 vs 5	9.57%	9.57%	9.57%	0.2419	9.57%	9.57%	9.57%	0.2348
Platform 4 vs 6	9.57%	9.57%	9.57%	0.2725	9.57%	9.57%	9.57%	0.2648
Platform 4 vs 7	9.57%	9.57%	9.57%	0.2834	9.57%	9.57%	9.57%	0.2761
Platform 4 vs 8	9.57%	9.57%	9.57%	0.2060	9.57%	9.57%	9.57%	0.2011
Platform 4 vs 9	9.57%	9.57%	9.57%	0.1746	9.57%	9.57%	9.57%	0.1705
Platform 4 vs 10	9.57%	9.57%	9.57%	0.2697	9.57%	9.57%	9.57%	0.2627
Platform 5 vs 6	23.40%	20.21%	9.57%	0.5838	21.28%	20.21%	9.57%	0.5737
Platform 5 vs 7	9.57%	9.57%	7.45%	0.4869	9.57%	9.57%	7.45%	0.4747
Platform 5 vs 8	9.57%	9.57%	9.57%	0.4271	9.57%	9.57%	9.57%	0.4169
Platform 5 vs 9	9.57%	9.57%	9.57%	0.3586	9.57%	9.57%	9.57%	0.3492
Platform 5 vs 10	9.57%	9.57%	9.57%	0.4459	9.57%	9.57%	9.57%	0.4341
Platform 6 vs 7	19.15%	10.64%	9.57%	0.5026	17.02%	10.64%	9.57%	0.4921
Platform 6 vs 8	10.64%	9.57%	9.57%	0.3876	10.64%	9.57%	9.57%	0.3787
Platform 6 vs 9	9.57%	9.57%	9.57%	0.4002	9.57%	9.57%	9.57%	0.3894
Platform 6 vs 10	9.57%	9.57%	9.57%	0.5971	9.57%	9.57%	9.57%	0.5848
Platform 7 vs 8	9.57%	9.57%	9.57%	0.4727	9.57%	9.57%	9.57%	0.4617
Platform 7 vs 9	9.57%	9.57%	9.57%	0.3667	9.57%	9.57%	9.57%	0.3572
Platform 7 vs 10	10.64%	9.57%	9.57%	0.5844	10.64%	9.57%	9.57%	0.5732
Platform 8 vs 9	12.77%	11.70%	9.57%	0.4772	11.70%	11.70%	9.57%	0.4666
Platform 8 vs 10	13.83%	9.57%	9.57%	0.4716	12.77%	9.57%	9.57%	0.4625
Platform 9 vs 10	9.57%	9.57%	9.57%	0.3723	9.57%	9.57%	9.57%	0.3611
Overall	12.65%	11.23%	9.81%	0.3596	12.20%	11.23%	9.79%	0.3515

Note: percentage indicates the portion of features met the criteria.

Supplementary Table S6. Ten most inter-platform reproducible radiomics between each platform within the VUE images and the VMI_{70keV}.

Feature name	ICC	Feature name	CCC
VUE images			
firstorder_Minimum	0.9844	firstorder_Minimum	0.9834
firstorder_10Percentile	0.9824	firstorder_10Percentile	0.9813
firstorder_Mean	0.9803	firstorder_Mean	0.9790
firstorder_Median	0.9800	firstorder_Median	0.9788
firstorder_90Percentile	0.9780	firstorder_90Percentile	0.9767
firstorder_Maximum	0.9769	firstorder_Maximum	0.9755
firstorder_RootMeanSquared	0.9744	firstorder_RootMeanSquared	0.9729
firstorder_Energy	0.9307	firstorder_Energy	0.9270
firstorder_TotalEnergy	0.9307	firstorder_TotalEnergy	0.9270
glcm_ClusterTendency	0.4787	glcm_ClusterTendency	0.4671
VMI_{70keV} images			
firstorder_10Percentile	0.9977	firstorder_10Percentile	0.9976
firstorder_Mean	0.9974	firstorder_Mean	0.9972
firstorder_Median	0.9974	firstorder_Median	0.9972
firstorder_90Percentile	0.9969	firstorder_90Percentile	0.9966
firstorder_RootMeanSquared	0.9966	firstorder_RootMeanSquared	0.9964
firstorder_Maximum	0.9962	firstorder_Maximum	0.9959
firstorder_Minimum	0.9952	firstorder_Minimum	0.9949
firstorder_Energy	0.9906	firstorder_Energy	0.9900
firstorder_TotalEnergy	0.9906	firstorder_TotalEnergy	0.9900
gldm_DependenceEntropy	0.5372	gldm_DependenceEntropy	0.5237

Supplementary Table S7. Inter-platform reproducibility of fourteen important radiomics features between each platform within the VUE images and the VMI_{70keV}.

No.	Feature name	Feature family	ICC in VUE images	CCC in VUE images	ICC in VMI_{70keV} images	CCC in VMI_{70keV} images
3	Entropy	First order	0.4368	0.4235	0.4469	0.4345
19	Uniformity	First order	0.3410	0.3286	0.3791	0.3679
22	Cluster Shade	GLCM	0.0918	0.0948	0.0948	0.0938
40	Maximum Probability	GLCM	0.1826	0.1747	0.2615	0.2520
52	Large Dependence High Gray Level Emphasis	GLDM	0.2338	0.2275	0.2166	0.2099
63	Long Run High Gray Level Emphasis	GLRLM	0.1999	0.1938	0.1759	0.1706
64	Long Run Low Gray Level Emphasis	GLRLM	0.1069	0.1030	0.2471	0.2398
65	Low Gray Level Run Emphasis	GLRLM	0.2003	0.1933	0.3611	0.3504
72	Short Run High Gray Level Emphasis	GLRLM	0.2573	0.2489	0.3046	0.2942
73	Short Run Low Gray Level Emphasis	GLRLM	0.2322	0.2240	0.3908	0.3793
90	Busyness	NGTDM	0.2421	0.2319	0.3050	0.2941
91	Coarseness	NGTDM	0.1297	0.1246	0.2227	0.2152
93	Contrast	NGTDM	0.1280	0.1229	0.1454	0.1391
94	Strength	NGTDM	0.2979	0.2890	0.2873	0.2776

Note: No. indicates the number of features in the Pyradiomics. Presents as average of ICC valves and average of ICC values of 45 comparisons. GLCM = gray-level co-occurrence matrix, GLDM = gray-level dependence matrix, GLRLM = gray-level run-length matrix, GLSZM = gray-level size zone matrix, NGTDM = neighborhood gray-tone difference matrix.

Supplementary Table S8. CT number values per ROI of each platform.

HU	Platform 1	Platform 2	Platform 3	Platform 4	Platform 5	Platform 6	Platform 7	Platform 8	Platform 9	Platform 10
VUE images										
ROI 1	868.49	776.18	885.36	790.10	1212.07	954.94	842.63	920.34	942.53	877.97
ROI 2	176.94	140.45	183.86	147.01	273.65	173.28	174.98	193.33	198.42	185.07
ROI 3	473.96	428.61	486.33	439.44	637.41	514.13	466.46	498.19	507.06	498.95
ROI 4	345.67	274.48	357.98	286.50	549.50	358.58	344.64	395.56	401.22	378.70
ROI 5	231.35	201.93	237.45	209.53	325.69	240.16	205.66	244.10	253.49	238.15
ROI 6	48.07	37.84	50.14	40.76	74.10	47.00	27.29	45.79	52.29	47.25
ROI 7	1308.33	1164.81	1328.21	1185.82	1909.52	1471.77	1300.06	1402.25	1443.90	1291.12
ROI 8	120.46	94.68	124.00	98.58	178.99	112.62	121.54	124.51	132.25	122.68
ROI 9	19.82	26.75	15.61	24.18	8.46	26.80	17.91	7.52	21.74	27.90
ROI 10	-87.46	-81.55	-89.50	-84.38	-105.18	-87.48	-82.93	-99.07	-94.23	-78.14
ROI 11	221.50	194.11	221.59	194.72	307.30	227.13	213.17	230.41	240.40	243.08
ROI 12	0.19	-0.65	-1.82	-2.70	4.51	2.97	-16.36	-6.39	1.97	13.43
ROI 13	-531.87	-534.33	-535.38	-538.55	-536.80	-530.64	-516.69	-533.16	-538.71	-539.62
ROI 14	75.97	75.14	73.05	72.27	75.55	77.32	65.95	67.10	78.04	78.69
ROI 15	-45.71	-42.96	-50.19	-46.60	-58.05	-46.90	-42.47	-57.22	-50.99	-38.38
ROI 16	64.84	52.39	61.67	49.16	85.62	60.07	53.17	58.08	69.30	79.03
VMI_{70keV}										
ROI 1	910.44	898.00	930.28	926.00	985.12	964.79	842.25	926.93	941.55	955.21
ROI 2	193.85	191.13	201.95	202.93	186.82	176.30	172.11	194.57	197.68	200.63
ROI 3	494.66	488.72	508.88	507.96	528.07	518.78	466.64	498.32	506.51	532.14
ROI 4	379.13	373.71	393.13	393.86	378.37	363.88	344.16	394.27	400.44	440.67
ROI 5	244.53	240.26	251.17	252.30	254.22	243.03	205.16	242.89	253.05	252.20
ROI 6	51.97	49.28	54.23	55.15	52.63	47.79	29.30	45.30	52.29	51.82
ROI 7	1372.55	1350.07	1395.60	1391.33	1529.65	1488.78	1301.24	1413.38	1443.99	1402.06
ROI 8	131.70	128.24	136.26	136.17	123.92	114.74	122.76	127.58	131.49	136.51
ROI 9	16.40	16.69	12.32	16.08	22.90	26.38	18.38	8.63	18.58	38.07
ROI 10	-90.19	-89.33	-92.09	-92.12	-90.25	-88.14	-82.37	-98.62	-94.63	-80.90
ROI 11	234.96	235.47	235.70	240.33	237.93	230.17	213.56	230.59	240.39	274.16
ROI 12	0.41	0.68	-1.40	-0.41	2.23	3.09	-19.52	-7.88	0.05	23.74
ROI 13	-531.03	-530.19	-534.78	-537.07	-535.52	-530.97	-517.14	-534.91	-538.37	-546.48
ROI 14	76.20	76.63	73.25	75.69	75.85	77.43	66.85	67.77	77.92	88.95
ROI 15	-47.11	-46.45	-51.78	-49.82	-49.60	-47.24	-43.53	-55.71	-52.96	-37.87
ROI 16	70.52	70.98	67.78	70.90	61.32	60.79	52.38	60.03	68.49	92.58

Supplementary Figure S1. Heatmap of Test-retest repeatability.

Percentages indicated the portion of feature scan-rescan measurements that did not exceed the 95% limits of agreement. GLCM = gray-level co-occurrence matrix, GLDM = gray-level dependence matrix, GLRLM = gray-level run-length matrix, GLSZM = gray-level size zone matrix, NGTDM = neighborhood gray-tone difference matrix.

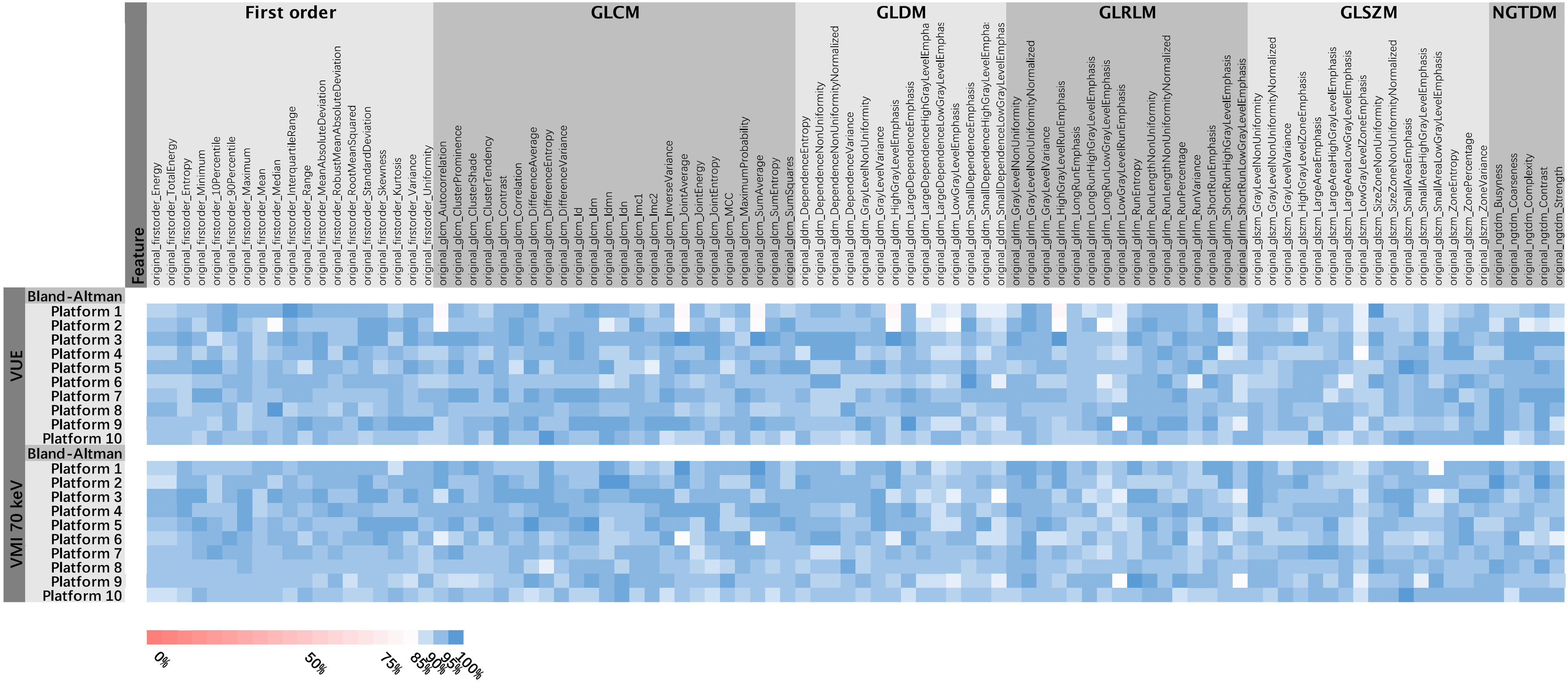

Supplementary Figure S2. Inter-platform reproducibility of radiomics between each platform within the VUE images and the VMI_{70keV}.

(A) VUE images. Left and right graphs showed percentages of radiomic features that were deemed as inter-platform reproducible between each platform per ICC > 0.90 and CCC > 0.90 within the VUE images, respectively, according to 45 comparisons.

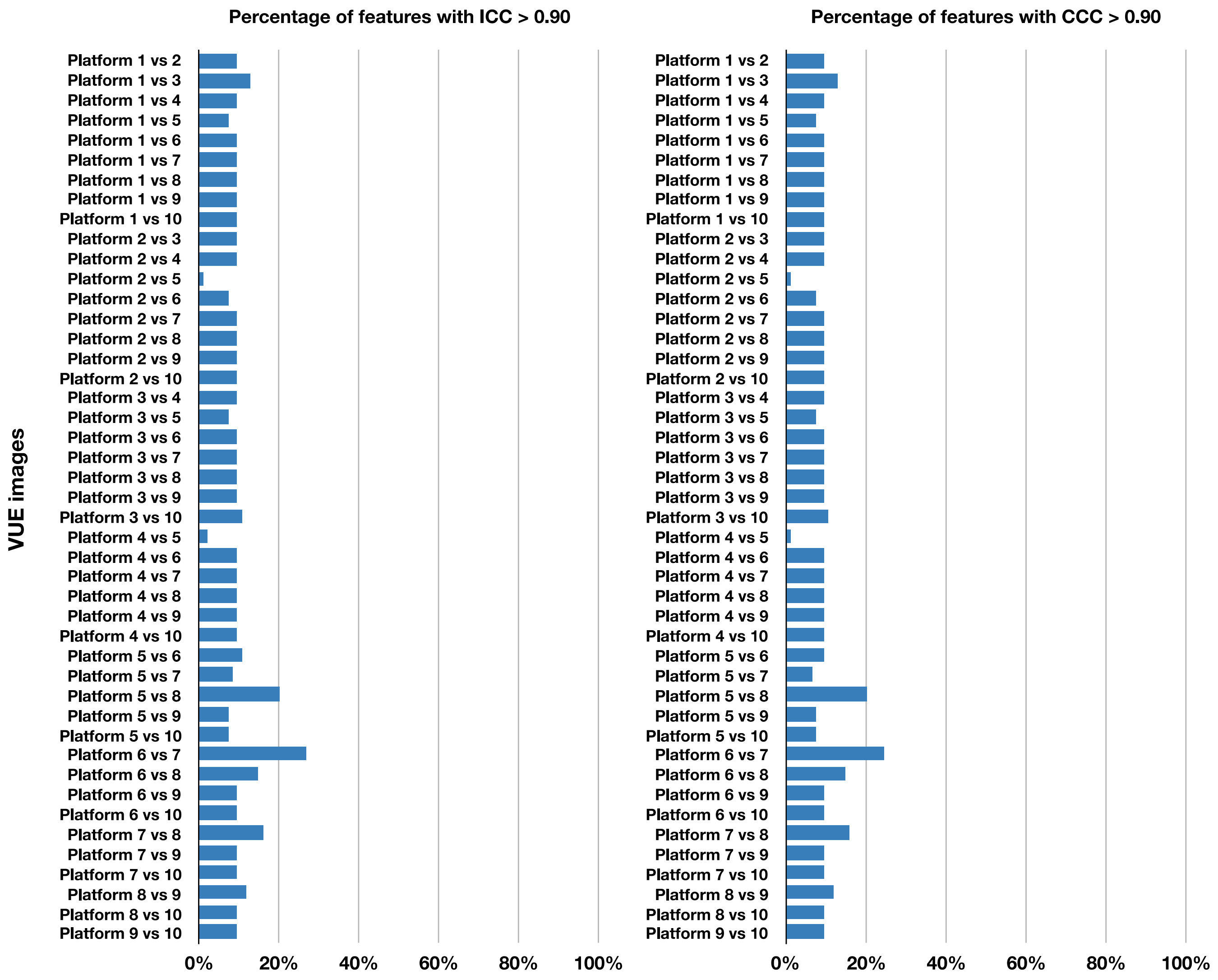

Supplementary Figure S2. Inter-platform reproducibility of radiomics between each platform within the VUE images and the VMI_{70keV}.

(B) VMI_{70keV}. Left and right graphs showed percentages of radiomic features that were deemed as inter-platform reproducible between each platform per ICC > 0.90 and CCC > 0.90 within the VMI_{70keV}, respectively, according to 45 comparisons.

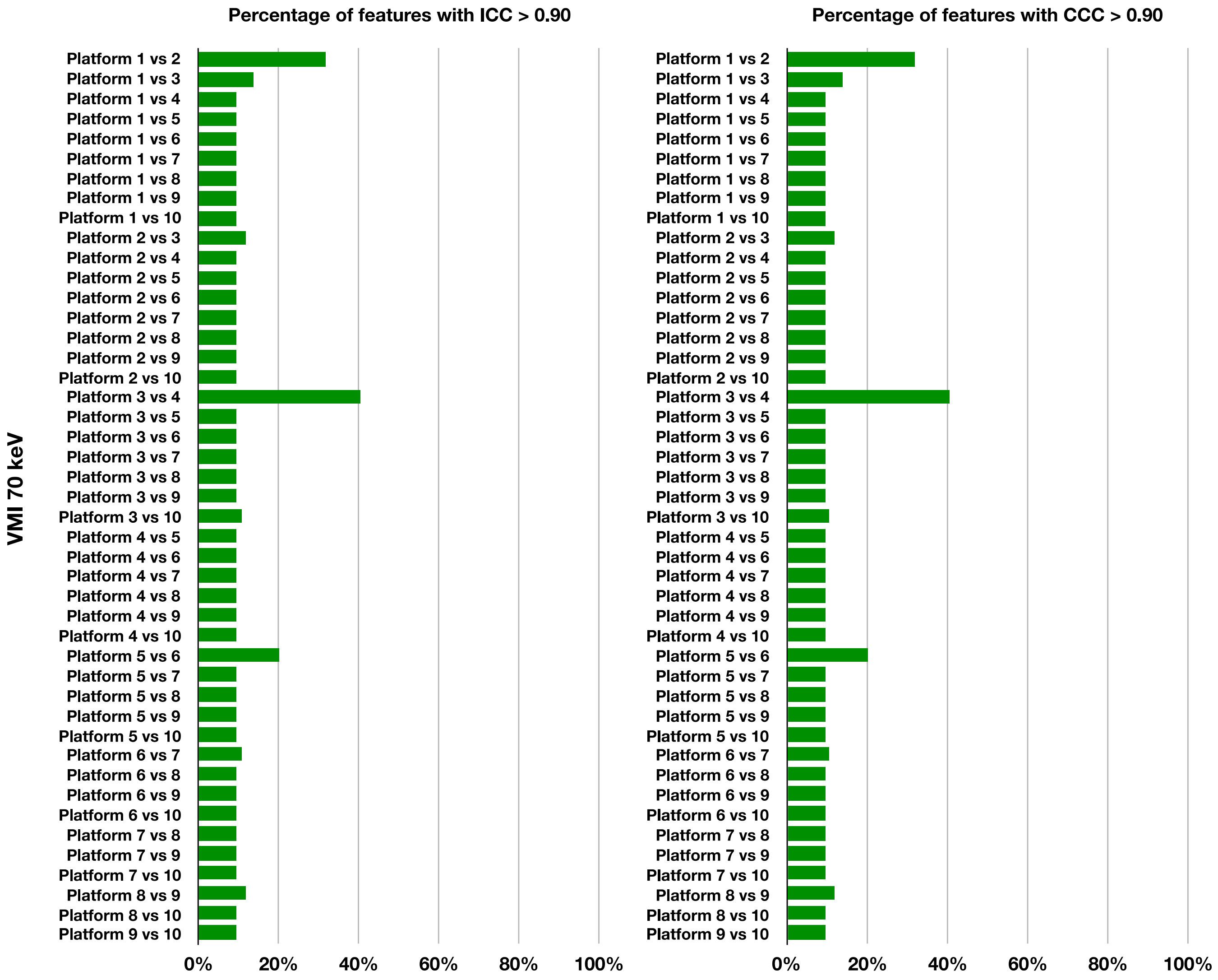

Supplementary Figure S3. Heatmap of inter-platform reproducibility of radiomics between each platform within the VUE images and the VMI70keV.

(A) VUE images. Number indicated ICC values and CCC values**.** GLCM = gray-level co-occurrence matrix, GLDM = gray-level dependence matrix, GLRLM = gray-level run-length matrix, GLSZM = gray-level size zone matrix, NGTDM = neighborhood gray-tone difference matrix.

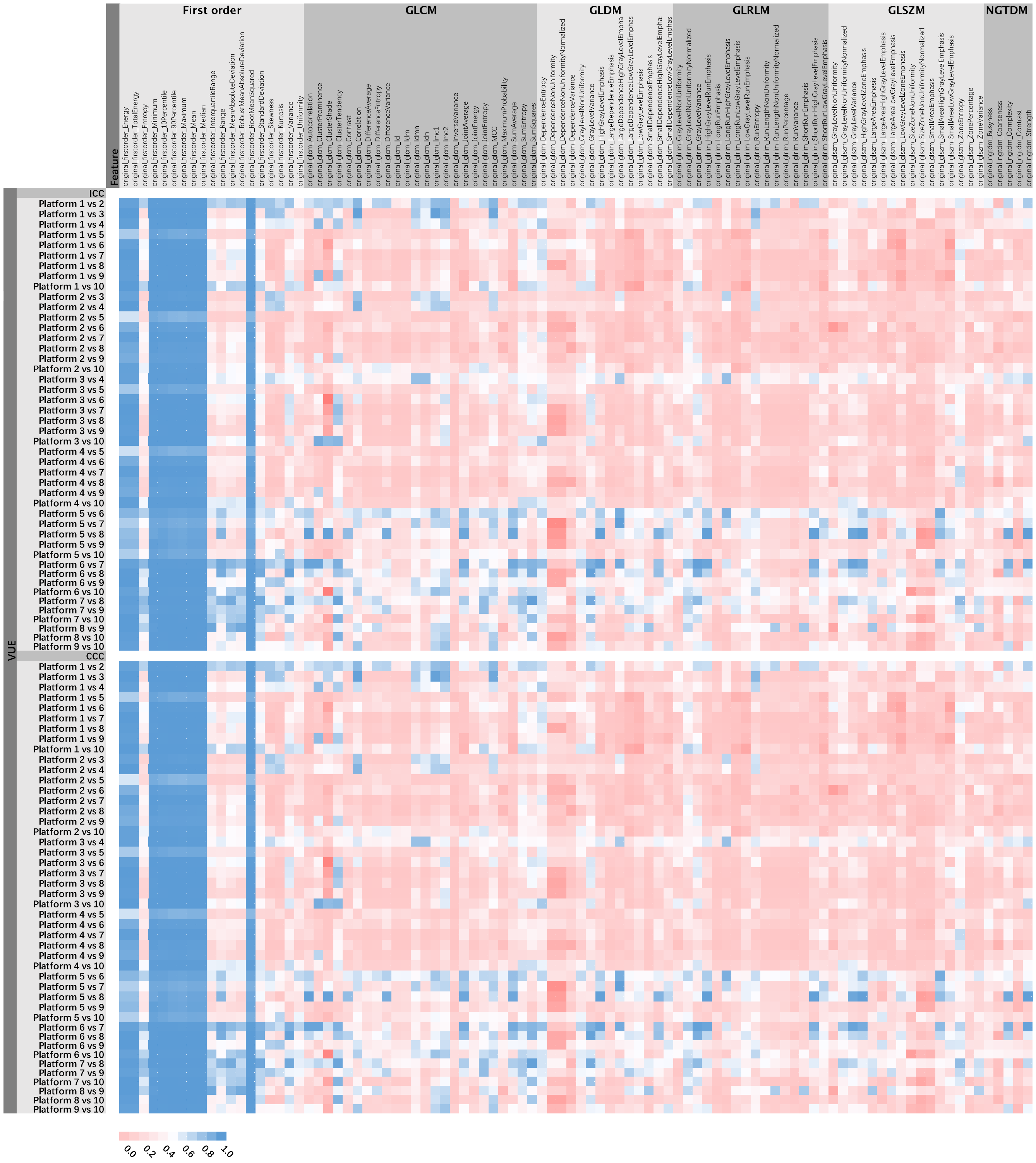

Supplementary Figure S3. Heatmap of inter-platform reproducibility of radiomics between each platform within the VUE images and the VMI_{70keV}.

(B) VMI_{70keV}. Number indicated ICC values and CCC values. GLCM = gray-level co-occurrence matrix, GLDM = gray-level dependence matrix, GLRLM = gray-level run-length matrix, GLSZM = gray-level size zone matrix, NGTDM = neighborhood gray-tone difference matrix.

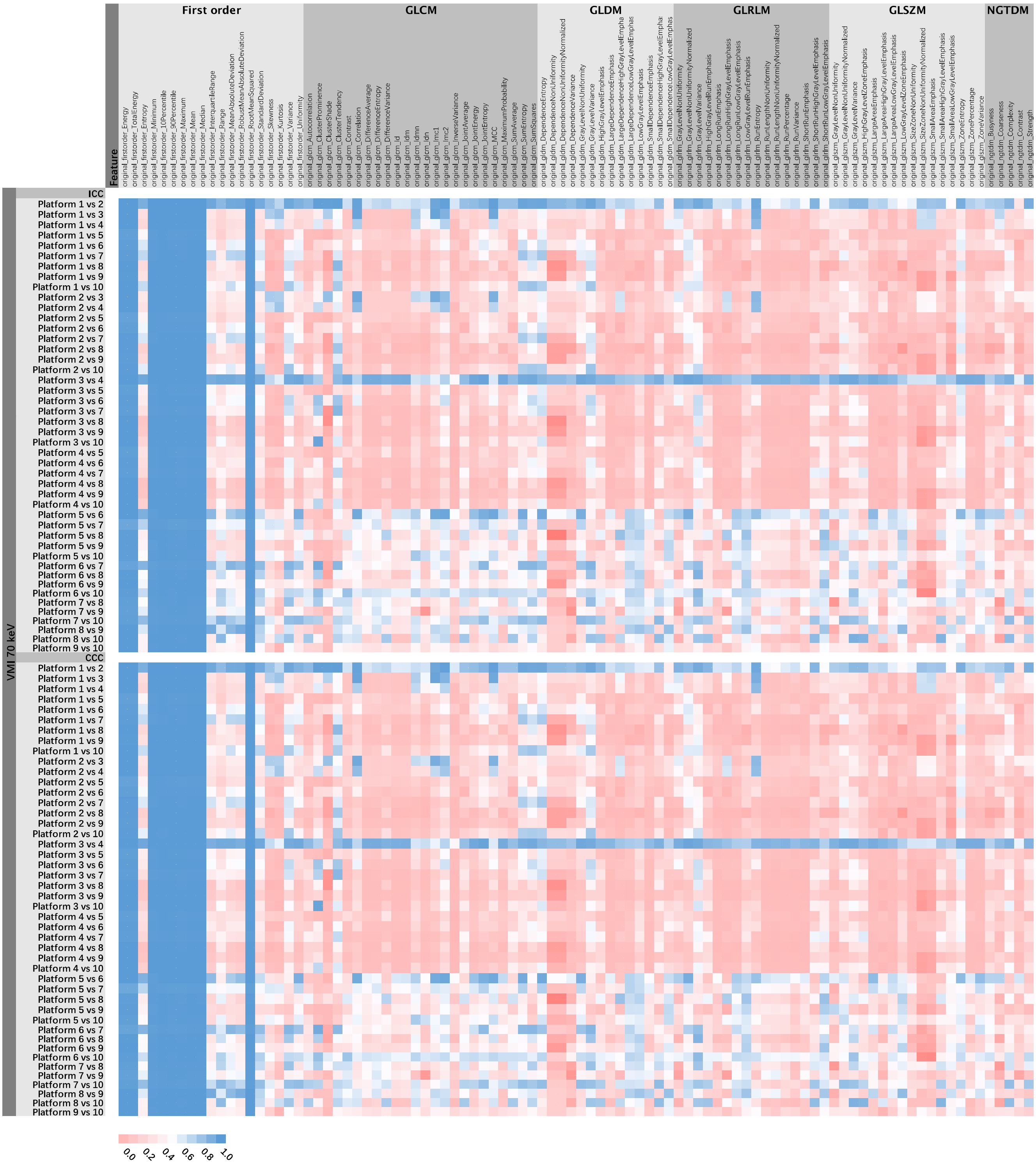

Supplementary Figure S4. Correlation of inter-platform CT number reproducibility and radiomics reproducibility.

(A) Inter-reproducibility within all The VUE images and the VMI_{70keV} using CV and QCD.

The dots indicated ROIs. The ROI 12 was excluded since it was considered as outlier; therefore, there were 15 ROIs in each correlation analysis. The correlation between CT number reproducibility of (CV values and QCD values) and percentage of radiomics features that met the criteria of reproducibility (CV < 10% and QCD < 10%) was quantitatively estimated by Spearman correlation analysis due to nonnormal distribution of data.

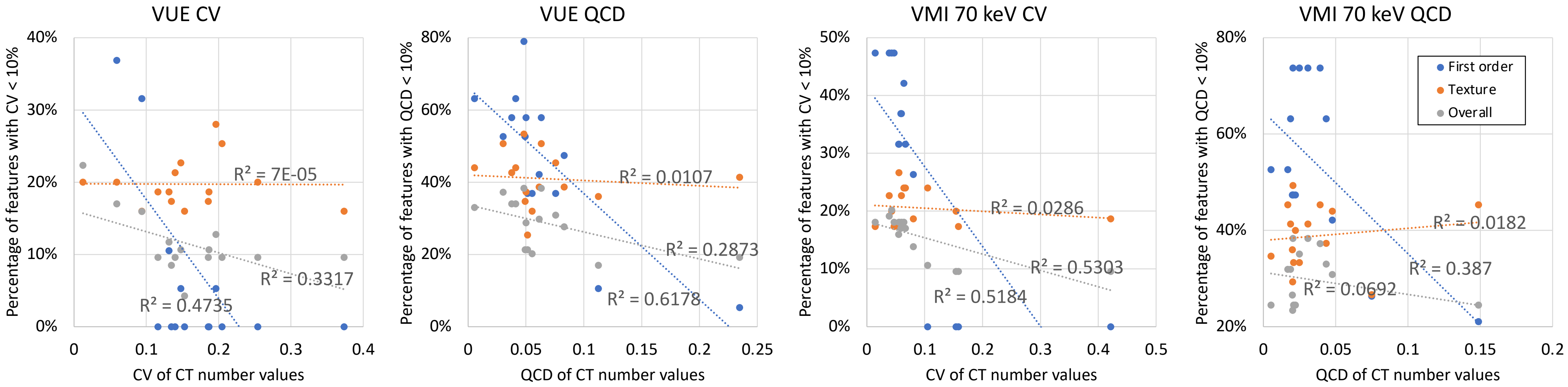

Supplementary Figure S4. Correlation of inter-platform CT number reproducibility and radiomics reproducibility.

(B) Inter-reproducibility between each platform within the VUE images and the VMI_{70keV} using ICC and CCC.

The dots indicated each platform. There were 45 comparisons in each correlation analysis. The correlation between CT number reproducibility of (ICC values and CCC values) and percentage of radiomics features met the criteria of reproducibility (ICC > 0.90 and CCC > 0.90) was quantitatively estimated by Spearman correlation analysis due to nonnormal distribution of data.

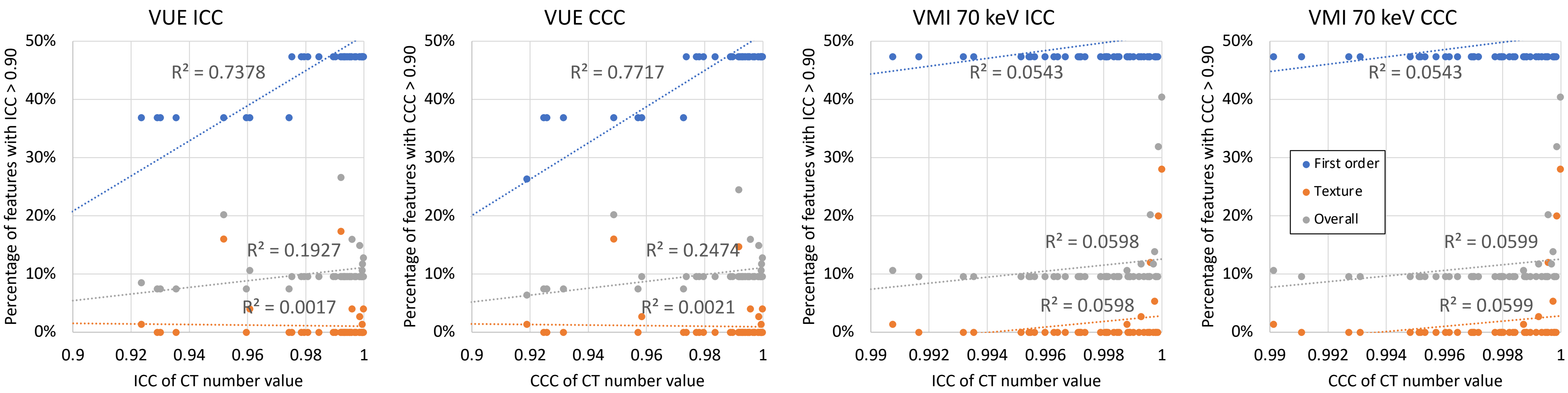

Supplement: Supplementary file 1 — Additional file 1. Supplementay Materials. [file 13244_2023_1426_MOESM1_ESM.pdf]
